# The tobacco phosphatidylethanolamine-binding protein NtFT4 increases the lifespan of *Drosophila melanogaster* by interacting with the proteostasis network

**DOI:** 10.18632/aging.204005

**Published:** 2022-04-08

**Authors:** Philip Känel, Gundula A. Noll, Katrin Schroedter, Elke Naffin, Julia Kronenberg, Franziska Busswinkel, Richard M. Twyman, Christian Klämbt, Dirk Prüfer

**Affiliations:** 1 Fraunhofer Institute for Molecular Biology and Applied Ecology IME, Münster, Germany; 2 Institute of Plant Biology and Biotechnology, University of Münster, Münster, Germany; 3 Institute of Neuro- and Behavioral Biology, University of Münster, Münster, Germany; 4 TRM Ltd, Scarborough, United Kingdom

**Keywords:** aging, proteostasis, heat shock proteins, chaperone, locomotor activity

## Abstract

Proteostasis reflects the well-balanced synthesis, trafficking and degradation of cellular proteins. This is a fundamental aspect of the dynamic cellular proteome, which integrates multiple signaling pathways, but it becomes increasingly error-prone during aging. Phosphatidylethanolamine-binding proteins (PEBPs) are highly conserved regulators of signaling networks and could therefore affect aging-related processes. To test this hypothesis, we expressed PEPBs in a heterologous context to determine their ectopic activity. We found that heterologous expression of the tobacco (*Nicotiana tabacum*) PEBP NtFT4 in *Drosophila melanogaster* significantly increased the lifespan of adult flies and reduced age-related locomotor decline. Similarly, overexpression of the Drosophila ortholog CG7054 increased longevity, whereas its suppression by RNA interference had the opposite effect. In tobacco, NtFT4 acts as a floral regulator by integrating environmental and intrinsic stimuli to promote the transition to reproductive growth. In Drosophila, NtFT4 engaged distinct targets related to proteostasis, such as HSP26. In older flies, it also prolonged *Hsp26* gene expression, which promotes longevity by maintaining protein integrity. In NtFT4-transgenic flies, we identified deregulated genes encoding proteases that may contribute to proteome stability at equilibrium. Our results demonstrate that the expression of NtFT4 influences multiple aspects of the proteome maintenance system via both physical interactions and transcriptional regulation, potentially explaining the aging-related phenotypes we observed.

## INTRODUCTION

Aging is characterized by the progressive disruption of cellular functions due to the accumulation of damaged DNA and proteins, which leads to the loss of homeostasis. Several proteins and signaling pathways control cellular homeostasis, including phosphatidylethanolamine-binding proteins (PEBPs), which are found in both animals and plants. In mammals, there are two conserved PEBPs (PEBP1-like and PEBP4-like) that integrate multiple signaling pathways to regulate cell behavior [1–3]. PEBPs have not been associated directly with aging at the organism level, but the dysregulation of PEBP expression correlates with tissue and organ degeneration. For example, the two human PEBPs are associated with several age-related, degenerative diseases, including diabetic nephropathy, Alzheimer’s disease, and various cancers [4–9]. *PEBP4* expression is tightly regulated in healthy tissues, whereas *PEBP1* (also known as Raf kinase inhibitory protein, RKIP) is ubiquitously expressed, and its activity is mainly regulated by PKC-mediated phosphorylation at S153. A role in lipid or phospholipid metabolism was proposed for these proteins based on their ability to bind phosphatidylethanolamine or phosphatidylcholine, but this aspect has received little attention following the discovery that the molecular basis of PEBP pathogenicity mostly reflects their ability to inhibit protein kinases [10].

Eight *Drosophila melanogaster* genes encode PEBP-like proteins that are structurally similar to human RKIP (*Pebp1*, *CG10298*, *CG7054*, *CG6180*, *CG17919*, *a5*, *CG17917* and *CG30060*; Supplementary Figure 1). Some are expressed preferentially in certain tissues (*Pebp1* in the midgut; *CG10298, CG17917* and *CG30060* in the testis; and *a5* in the adult head) whereas *CG7054*, *CG6180* and *CG17919* are expressed ubiquitously [11]. Pebp1 was recently shown to be important for the regenerative capacity of the intestinal stem cell (ISC) niche because its suppression led to accelerated ISC proliferation promoted by the loss of enterocytes, whose survival relies on *Pebp1* expression. Declining *Pebp1* expression, as also observed during aging, was accompanied by the loss of its ability to inhibit extracellular signal-regulated kinase (ERK) activity and the tight regulation of EGFR/ERK signaling [12]. In addition, PEBP-like proteins may control innate immunity but their molecular functions in Drosophila remain largely unclear [13–16].

Three distinct PEBP subclades have evolved in flowering plants, related to the floral regulators TERMINAL FLOWER 1 (TFL1), FLOWERING LOCUS T (FT), and MOTHER OF FT AND TFL1 (MFT), respectively [17]. The best characterized plant PEBPs are the TFL1-like and FT-like proteins, the latter being of particular interest due to their further functional diversification [18–23]. FT-like proteins with opposing roles during development are involved in the formation of storage organs, such as potato tubers, but also during the floral transition. In our experiments, we tested two tobacco PEBPs comprising a representative floral activator (NtFT4) and floral repressor (NtFT2) from the FT-like subclade [18]. Whereas the different functions of human PEBPs are associated with overtly distinct structures, single amino acid exchanges in plants are sufficient to convert a floral activator into a floral repressor [24]. The ability for such subtle differences to define functionality, and the consistent lack of the typical C-terminal helix, are unique properties of plant PEBPs [25, 26].

To investigate the functions of PEBPs in more detail, we undertook interspecies analysis and determined the molecular, cellular and organism-level effects of animal PEBPs expressed in Arabidopsis (*Arabidopsis thaliana*) and tobacco (*Nicotiana tabacum*) and plant PEBPs expressed in Drosophila. The functions of animal PEBPs in plants were assessed by investigating their interaction with canonical partners of FT-like proteins and by the overexpression of different PEBPs. We selected the best-characterized human PEBPs (*RKIP* and *hPEBP4*) and Drosophila PEBPs (*Pebp1* and *CG7054*) for the stable transformation of the two model plants and subsequent phenotypic analysis. In a reciprocal experiment, we used the Gal4 system to individually express two closely-related but functionally distinct plant PEBPs (*NtFT2* and *NtFT4*) as well as their closest Drosophila homolog (*CG7054*) in Drosophila. Although the expression of animal PEBPs in plants had no significant effect on flowering time, we were able to confirm molecular interactions with the anticipated endogenous binding partners. In contrast, the expression of plant PEBPs in Drosophila increased the adult fly lifespan by up to one third, whereas the silencing of the endogenous PEBP CG7054 reduced longevity. This observation correlates with the ability of NtFT4 to promote the expression of the small heat shock genes *Hsp26* and *Hsp27* in older flies and its ability to interact with the HSP26 protein. Thus, our results indicate that PEBPs extend the activity of the proteome maintenance system.

## RESULTS

### The expression of animal PEBPs in plants has no effect on floral transition or growth

**Figure 1:**
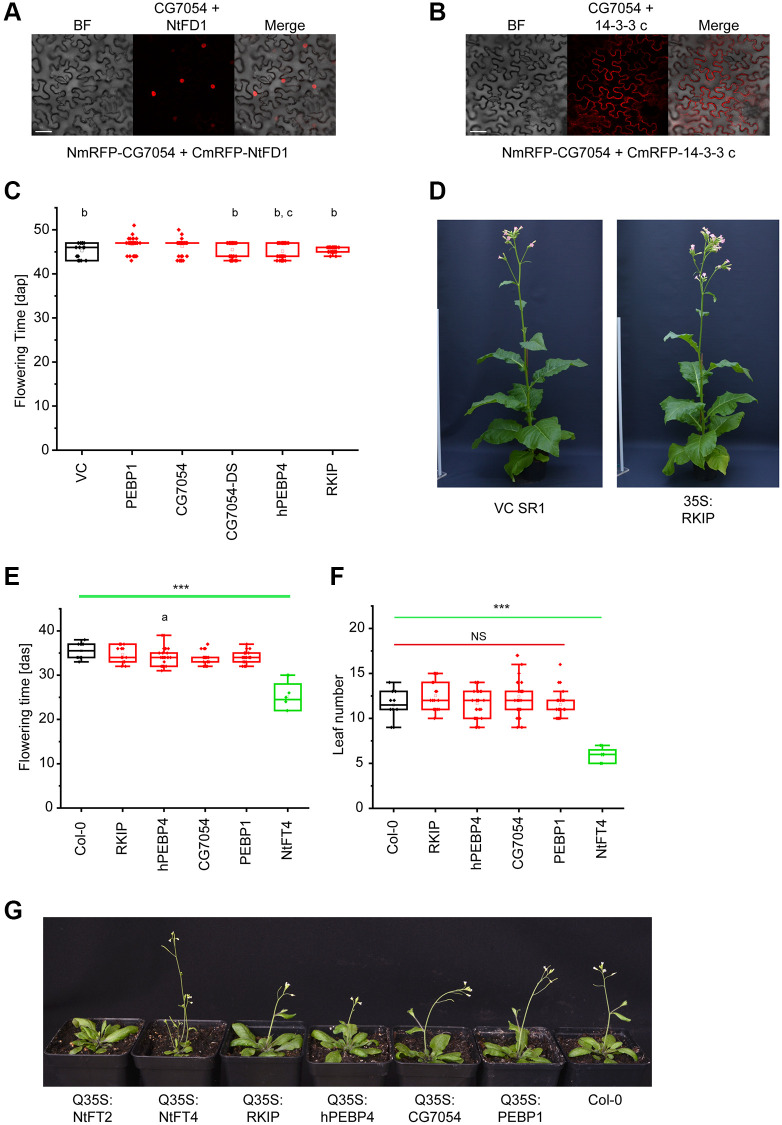
**Expression of animal PEBPs in tobacco and Arabidopsis.** (**A**) Bimolecular fluorescence complementation (BiFC) in infiltrated *Nicotiana benthamiana* leaves, representatively showing the interaction between Drosophila PEBP (NmRFP-CG7054) and NtFD1 (CmRFP-NtFD1). (**B**) BiFC representatively showing the interaction between Drosophila PEBP (NmRFP-CG7054) and tobacco 14-3-3 c (CmRFP-14-3-3 c). Scale bar = 50 μm. (**C**) Flowering time of tobacco lines expressing PEBP1, CG7054, CG7054-DS, RKIP or hPEBP4 under the control of the cauliflower mosaic virus 35S promoter. Abbreviation: VC: vector control. Flowering time was measured under long-day (LD) conditions in days after potting (dap). Data are means ± SEM, *n* = 50 (PEBP1, CG7054, CG7054-DS, RKIP and hPEBP4), *n* = 10 (VC). Significance was tested by one-way ANOVA and Tukey’s *post hoc* test (*b* significant compared with PEBP1, *c* significant compared with CG7054, all other comparisons non-significant). (**D**) Representative image of a transgenic tobacco plant expressing RKIP compared with the VC. Flowering time (**E**) and rosette leaf number at the onset of flowering (**F**) of transgenic Arabidopsis lines expressing RKIP, hPEBP4, CG7054, PEBP1 or the floral inducer NtFT4 under the control of the quadruple cauliflower 35S promoter. Col-0 = wild type *A. thaliana* Col-0 ecotype used for transformation. Flowering time was measured under LD conditions in days after seeding (das). Data are means ± SEM, *n* = 30 (CG7054, CG19594), *n* = 29 (hPEBP4), *n* = 19 (RKIP), *n* = 10 (Col-0), *n* = 8 (NtFT4); ^****^p < 0.001 in all pairwise comparisons with NtFT4 (*a* significant compared with Col-0 (*p* = 0.091) with all other comparisons being non-significant). Abbreviation: *NS*: no significant differences in any pairwise comparison. All *p*-values are provided in Supplementary Table 9. (**G**) Representative images of transgenic Arabidopsis plants expressing different PEBPs. Col-0 wild type plants (far right), and early flowering Q35-S:NtFT4 (left) and late flowering Q35-S:NtFT2 (far left) plants are shown in comparison with plants expressing the animal PEBPs.

The regulation of flowering time by FT-like proteins requires the binding of 14-3-3 scaffolding proteins to recruit specific bZIP transcription factors such as NtFD1 [18, 27]. We found that Drosophila CG7054 (which has the highest similarity to tobacco PEBPs) is also able to interact with tobacco 14-3-3 proteins and the transcription factor NtFD1 in *Nicotiana benthamiana* leaves, as revealed by bimolecular fluorescence complementation (BiFC) (Figure 1A, 1B). But despite these canonical interactions, the ubiquitous expression of CG7054 or other Drosophila or human PEBPs – PEBP1, a chimeric CG7054 carrying segments of NtFT4 (CG7054-DS, Supplementary Figure 2), RKIP and hPEBP4 – in Arabidopsis and tobacco had a negligible impact on flowering time (Figure 1C–1G).

We established stable transgenic lines expressing these PEBPs under the control of the strong cauliflower mosaic virus 35S promoter (35S) or the quadruple 35S promoter (Q35S) and selected independent lines with high PEBP expression levels for phenotyping. All tobacco plants expressing animal PEBPs flowered ~46 days after potting (dap), specifically hPEBP4 = 45.14 ± 0.24 dap and PEBP1 = 46.7 ± 0.20 dap, which was comparable to the vector control (45.3 ± 0.44 dap). The maximum delay was 1.4 days for PEBP1 (Figure 1C). In addition, ubiquitous expression of animal PEBPs did not cause any change in plant size or architecture (Figure 1D). In Arabidopsis, flowering times ranged from 32.7 ± 0.47 days after seeding (das) (hPEBP4) to 35.6 ± 0.57 das (RKIP) in lines expressing animal PEBPs, and were therefore comparable to the control (35.7 ± 0.61 das) and significantly later than flowering in the line expressing the floral activator NtFT4 (25.3 ± 0.61 das, Figure 1E).

The difference between animal and plant PEBPs was also very pronounced when comparing rosette leaf numbers at the onset of flowering (Figure 1F, 1G). NtFT4 expression significantly reduced the leaf number at this stage to 5.71 ± 0.29, whereas control plants (12.0 ± 0.62) and lines expressing animal PEBPs (PEBP4 = 11.71 ± 0.64, RKIP = 13.29 ± 0.61) had similar numbers of leaves. Some plants expressing the floral repressor NtFT2 did not flower by the end of the experiment (Figure 1G). Interaction with 14-3-3 proteins and the transcription factor FD therefore appears to be necessary, but not sufficient, for floral regulation.

### PEBPs increase the lifespan of Drosophila

**Figure 2:**
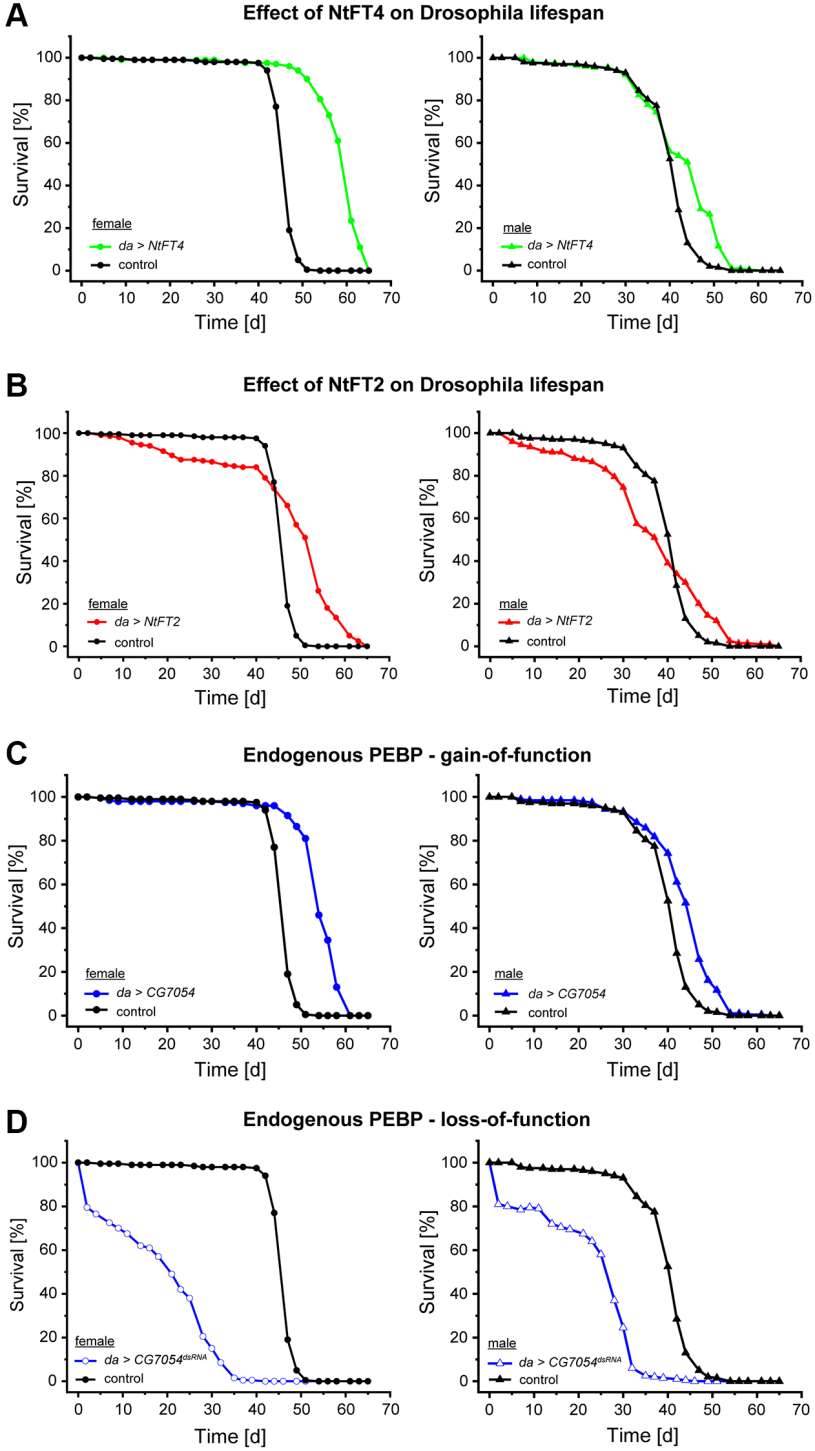
**Survival of Drosophila populations expressing NtFT4, NtFT2, CG7054 or CG7054^dsRNA^ under the control of the *daughterless* (*da*) promoter**. Survival curves of female (left) and male (right) flies in the filial generation after mating *UAS-NtFT4*, *UAS-NtFT2*, *UAS-CG7054* or *UASt-CG7054^dsRNA^* with the *da-Gal4* driver strain. (**A**, **B**) Effect on lifespan of flies constitutively expressing the floral inducer NtFT4 (**A**) or the floral repressor NtFT2 (**B**) compared with *da-Gal4* x Oregon-R (*n* = 200). (**C**, **D**) Effect on lifespan of flies constitutively expressing the Drosophila PEBP *CG7054* (**C**) or constitutively silencing *CG7054* after mating *UASt-CG7054^dsRNA^* with the *da-Gal4* driver strain (**D**) compared with *da-Gal4* x Oregon-R (*n* = 200). Median and mean lifespans and statistical evaluation are summarized in Table 1 (female flies) and Supplementary Table 1 (male flies).

In the reciprocal experiment, we investigated the impact of expressing tobacco PEBPs (NtFT4 or NtFT2) or Drosophila CG7054 on fly morphogenesis and aging. We prepared UAS-based expression constructs and used the φC31 system for integration into the landing site 86Fb to ensure comparable expression levels for each transgene [28]. All constructs were constitutively expressed using the *daughterless-Gal4* system (*da-Gal4*). The specific role of Drosophila PEBPs in aging has not been reported before, so we also silenced the *CG7054* gene by RNA interference (RNAi) and investigated the physiological effects. Longevity was determined in groups of 20 mated females or males for all lines (lifespan data for male flies are provided in Supplementary Table 1). Among all the overexpression lines, the ubiquitous expression of NtFT4 showed the strongest effect on longevity (Table 1, Figure 2A), increasing the lifespan of female flies by 29.8% (median lifespan NtFT4♀ = 61 days, control♀ = 47 days). The expression of CG7054 or NtFT2 increased the lifespan by 14.9% (median lifespan CG7054♀ = 54 days, NtFT2♀ = 54 days; Table 1, Figure 2B, 2C). However, analysis of the first quartile (25% of the NtFT2 population) based on Kaplan-Meier survival curves revealed early mortality (NtFT2♀ = 44 days, control♀ = 47 days) whereas the opposite was observed for flies expressing CG7054 or NtFT4, where the first quartile survived longer than control flies (CG7054♀ = 54 days, NtFT4♀ = 56 days). CG7054 and NtFT4 therefore conferred a degree of longevity, but NtFT4 extended the lifespan significantly further than CG7054 (Table 1). The knockdown of *CG7054* in muscle cells using *Mef2-Gal4* was previously shown to cause late pupal lethality [29]. We used the *da-Gal4* system to achieve *CG7054* knockdown in all cells, which caused 40% of the animals to die during late pupal stages (*n* = 748). The surviving adult flies expressing *CG7054^dsRNA^* had much shorter lifespans, reduced by 40.5% in males and 55.3% in females compared to controls (Figure 2D). In addition to the overall shorter lifespan, the knockdown of *CG7054* also caused approximately 20% of adult flies to die within two days (Table 1, Figure 2D).

**Table 1:** Survival of female flies with dysregulated PEBP expression ([*da-Gal4/UAS-CG7054*, *da-Gal4/UAS-NtFT2* or *da-Gal4/UAS-NtFT4*] or [*da-Gal4/UASt-CG7054^dsRNA^*]) compared to +/*da-Gal4* controls.

	**Median lifespan [d]**	**25% Estimate [d]**	**Mean lifespan [d]**	**Equality vs. control (χ^2^)**	**Equality vs. CG7054 (χ^2^)**	**Equality vs. NtFT2 (χ^2^)**
**Control**	47	47	46.19 (± 0.34)	−	−	−
**CG7054**	54	54	54.00 (± 0.57)	313.72 (*p* = 0)	−	−
**CG7054^dsRNA^**	21	7	18.91 (± 0.83)	447.62 (*p* = 0)	440.96 (*p* = 0)	−
**NtFT2**	54	44	47.66 (± 0.97)	94.87 (*p* = 0)	14.02 (*p* = 1.81 × 10^-4^)	−
**NTFT4**	61	56	58.50 (± 0.51)	371.28 (*p* = 0)	110.56 (*p* = 0)	119.98 (*p* = 0)

Median lifespans, 25% quartile estimates and mean lifespans were calculated based on Kaplan-Meier survival curves and χ^2^ and *p*-values were calculated using the Mantel-Cox method.

### PEBPs increase locomotor activity of Drosophila

**Figure 3:**
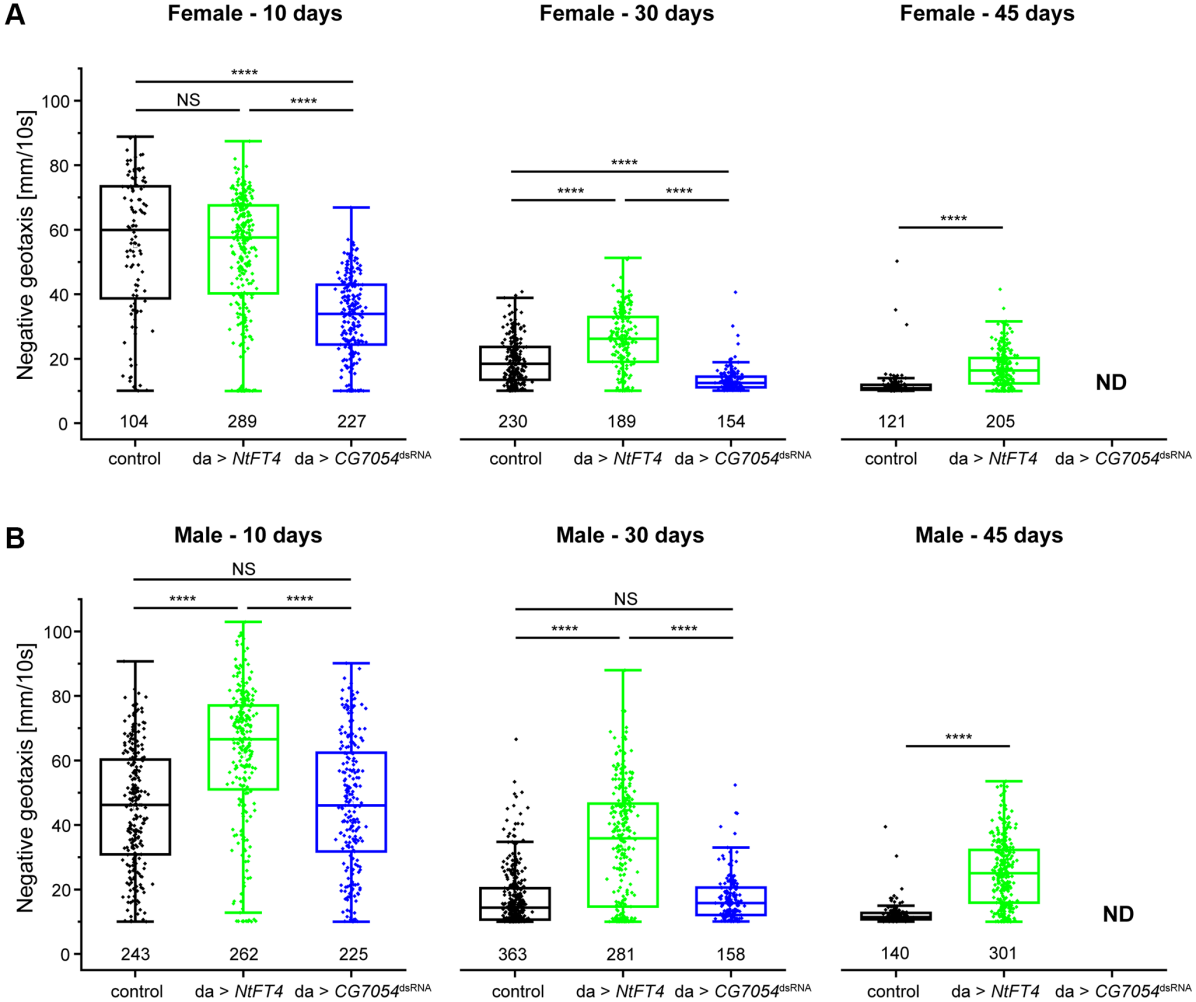
**Locomotor behavior of long-lived (*da > NtFT4*) or short-lived (*da > CG7054^dsRNA^*) Drosophila populations at different ages.** Rapid iterative negative geotaxis (RING) assay with virgin female (**A**) and male (**B**) flies at 10, 30 or 45 days old. The locomotor behavior was analyzed in the filial generation after mating Oregon-R (control, black), *UAS-NtFT4* (green) or *UASt-CG7054^dsRNA^* (blue) flies with the *da-Gal4* driver strain. Negative geotaxis was plotted as average velocity (mm/10 s) and was traced for all tracks traveled in the population (numbers below plots). Significance was tested by one-way ANOVA and Tukey’s *post hoc* test between control, *da > NtFT4* and *da > CG7054^dsRNA^* (^****^p < 0.001, Abbreviation: *NS*: not significant). All *p*-values are provided in Supplementary Table 9.

Old flies expressing CG7054 or NtFT4 showed higher rates of motility than similarly-aged control flies. To quantify locomotion of adult flies we employed the rapid iterative negative geotaxis (RING) assay to characterize age-related decline in the locomotor ability of flies climbing the side of a tube [30]. Control female flies always show greater motility than age-matched males, so we tested the two sexes separately. We compared flies expressing NtFT4 (long-lived) or *CG7054^dsRNA^* (short-lived) to controls at different ages (10, 30 and 45 days) although the short lifespan of the *CG7054^dsRNA^* flies prevented the tests of this genotype at 45 days (Figure 3). Female *CG7054^dsRNA^* flies showed a consistent locomotor decline compared to controls and NtFT4 flies regardless of age (−40.25% and −38.86% at 10 days old, −30.16% and −47.50% at 30 days old, compared to control and NtFT4 flies, respectively; Figure 3A). Interestingly, *CG7054^dsRNA^* expression did not affect the locomotor activity of male flies, regardless of their age (Figure 3B). In contrast, the NtFT4 expression increased locomotor activity in male flies of all ages compared to controls (+36.09% at 10 days old, +97.37% at 30 days old, +105.68% at 45 days old; Figure 3B). In addition, male *da > NtFT4* flies were even more active at 45 days old than the control flies at 30 days old based on the velocity of negative geotaxis (NtFT4♂ 45 days = 2.54 mm/s, control♂ 30 days = 1.71 mm/s, *p* = 7.32 × 10^−9^). In young female flies, NtFT4 expression had no effect on locomotor activity, but it increased the locomotor activity of old females (+33.02% at 30 days old, +43.35% at 45 days old, compared to controls; Figure 3A). At this stage, old *da > NtFT4* females showed locomotion comparable to control flies 15 days younger (NtFT4♀ 45 days = 1.71 mm/s, control♀ 30 days = 1.92 mm/s, *p* = 0.64).

### Plant and animal PEBPs differ in stability and subcellular localization

In Drosophila, the different PEBPs were expressed from the same genomic locus suggesting that variation in expression levels should not account for the observed differences. To evaluate protein stability as a factor, we transiently expressed the different PEBPs with a hemagglutinin (HA) tag in Drosophila S2 and human embryonic kidney (HEK) 293T cells and compared mRNA and protein levels. In both cell lines, the plant PEBPs were less abundant than their fruit fly counterparts, particularly when comparing NtFT4 and CG7054 (Supplementary Figure 3). Although *HA-NtFT4* and *HA-CG7054* mRNA were expressed at comparable levels, only HA-CG7054 was detected in the protein extracts (Supplementary Figure 3A). Green fluorescent protein fusions of the tobacco FT-like proteins (HA-EGFP-NtFT4 and HA-EGFP-NtFT2) appeared more stable than HA-NtFT4 and HA-NtFT2 (Supplementary Figure 3B). As shown above for the HA-tagged constructs, the HA-EGFP-CG7054 protein accumulated to higher levels than HA-EGFP-NtFT4, although *HA-EGFP-NtFT4* mRNA was more abundant than *HA-EGFP-CG7054* mRNA (19.03 ± 1.5 vs. 5.95 ± 0.25; Supplementary Figure 3C). These data suggest there is no correlation between longevity and the abundance of PEBPs.

Interestingly, whereas the fly PEBPs CG7054, PEBP1 and CG10298 were uniformly located in all cellular compartments in HEK-293T and S2 cells, NtFT4 and NtFT2 were enriched in nuclear speckles (Supplementary Figure 3D). The distinctive nuclear localization of NtFT4 and NtFT2 was also found for HA-tagged NtFT proteins in Drosophila fat body cells (Supplementary Figure 3E). The distinct subcellular localization of NtFT4 and NtFT2 compared to CG7054 and PEBP1 may indicate a specific function in the nucleus. The NtFT2 and NtFT4 peptide sequences do not contain a nuclear localization signal to explain their accumulation (Supplementary Figure 4A). In plants, FT-like proteins translocate to the nucleus when they interact with FD-like bZIP transcription factors, and a similar mechanism may therefore operate in Drosophila cells.

### The NtFT4 interactome reflects its multifunctional role

To determine how NtFT4 affects longevity, we set out to identify its interaction partners in Drosophila using a yeast two-hybrid (Y2H) library and mass spectrometry following co-immunoprecipitation from extracts of transiently transfected S2 cells expressing HA-EGFP-tagged NtFT4. In the former case, we used a Drosophila normalized cDNA library to ensure the detection of rare interactions. Because the NtFT4 fusion with the DNA-binding domain of Gal4 (Gal4^BD^) caused auto-activation, we used the related NtFT2 protein as the initial bait. NtFT2 and NtFT4 share 70.2% amino acid sequence identity and they have similar predicted structures (Supplementary Figure 4B–4D). Moreover, NtFT2 overexpression increases longevity to the same extent as CG7054 (Table 1). We isolated 72 colonies from the cDNA library on selective medium. Sequencing and subsequent cloning of the full coding sequences followed by re-analysis in a drop test confirmed interactions between NtFT2 and nine Drosophila proteins (Supplementary Figure 5A). The interactions with CG6523, CG7220, CKIIα-i3, mRpL44, RHEB and YIPPEE were confirmed using BiFC assays (Supplementary Figure 5B), whereas the interactions with ACT42A, CG31644 and 4E-T remain uncertain because they were not verified in the Y2H drop test (ACT24A) or by BiFC (CG31644, 4E-T). The unconfirmed interactions in Y2H drop tests are shown in Supplementary Figure 6. Further BiFC experiments revealed that six of these initial candidates (ACT42A, CG6523, CG7220, CKIIα-i3, mRpL44 and RHEB) also interact with NtFT4 and CG7054. Interestingly, the YIPPEE protein was shown to interact with NtFT2 and CG7054 but not with NtFT4 (Supplementary Figure 5B).

**Figure 4:**
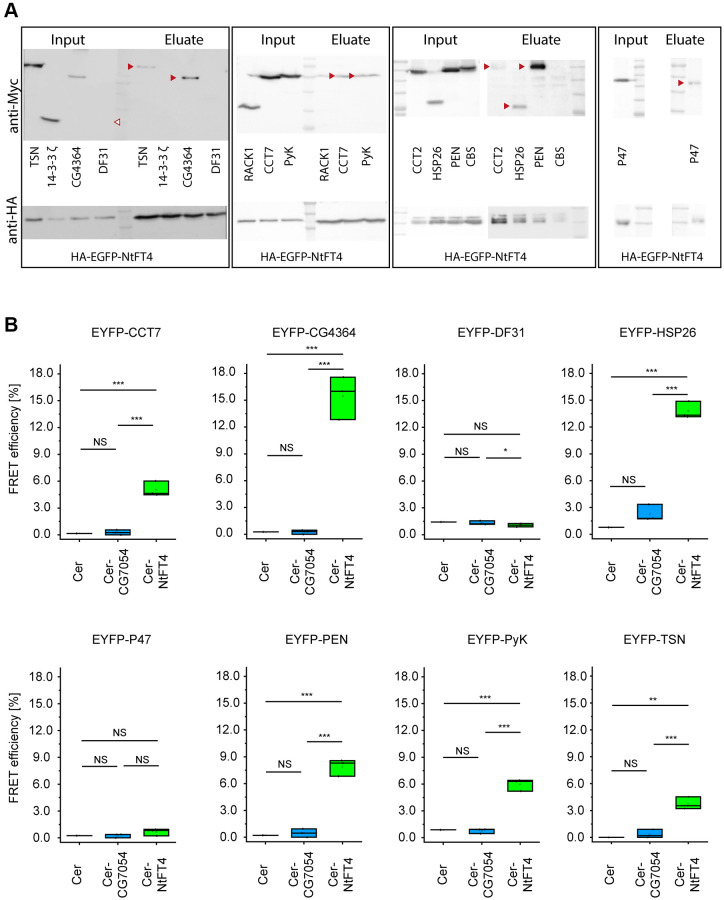
**Interaction partners of NtFT4 identified in immunoprecipitated protein complexes after transient expression in S2 cells.** The abundance of the interaction partners was confirmed by immunodetection using mouse anti-Myc (top) or rabbit anti-HA (bottom) antibodies in the extracts and successful precipitation with magnetic anti HA-beads was confirmed by the detection of HA-EGFP-NtFT4 in the eluates. (**A**) Western blots of extracts (Input) and eluates after co-immunoprecipitation (Eluate) following transient co-transfection of S2 cells with HA-EGFP-NtFT4 plus Myc-Tsn, Myc-14-3-3 ζ, Myc-CG4364, Myc-Df31, Myc-Rack1, Myc-CCT7, Myc-PyK, Myc-CCT2, Myc-Hsp26, Myc-Pen, Myc-Cbs or Myc-p47. Detection of co-immunoprecipitated proteins in the eluate is indicated by red arrowheads. Df31 was not detected in extracts under the mild conditions used for immunoprecipitation (empty arrowhead). (**B**) Analysis of FRET efficiency in co-transfected cells expressing the donors Cerulean (Cer, negative control), Cer-NtFT4 or Cer-CG7054 plus the acceptors EYFP-CCT7, EYFP-CG4364, EYFP-Df31, EYFP-Hsp26, EXFP-p47, EYFP-Pen, EYFP-PyK or EYFP-Tsn by flow cytometry. Gating strategy and representative controls are shown in Supplementary Figure 8. Cer-NtFT4 and Cer-CG7054 were co-transfected in three independent triplicates (*n* = 3) and statistical significance was tested by one-sample *t*-test (^****^p < 0.001, ^***^p < 0.01, ^**^p < 0.05, ^*^p < 0.1, Abbreviation: *NS*: not significant).

To refine the list of interaction partners in a Drosophila cell model, we performed immunoprecipitation experiments using transiently transfected S2 cells expressing NtFT4 tagged with HA-EGFP (at the C-terminus or N-terminus) and used HA-EGFP as a reference. We were unable to detect HA-tagged NtFT4 in extracts of the transgenic flies, thus preventing *in vivo* interaction assays. The precipitates generated using HA-EGFP and HA-EGFP-NtFT4 (Supplementary Figure 7) were analyzed by LC-MS/MS. This revealed 23 putative NtFT4 interaction partners (Supplementary Table 2). Following the cell model, we confirmed the interactions between NtFT4 and CCT7, CG4364, HSP26, PEN, PyK and TSN by co-immunoprecipitation (Figure 4A) and fluorescence resonance energy transfer (FRET) analysis (Figure 4B). The gating strategy to quantify FRET efficiency in all experiments is shown in Supplementary Figure 8. Although we detected a FRET signal when CG7054 was combined with HSP26, PEN and TSN, these interactions were inconclusive and significantly weaker than the corresponding assays with NtFT4. EYFP-HSP26 achieved the following FRET efficiencies: CER-NtFT4 = 13.8%, CER-CG7054 = 2.3% and CER = 0.78%. When testing EYFP-PEN, the equivalent results were CER-NtFT4 = 7.9%, CER-CG7054 = 0.5% and CER = 0.2%. Finally with EYFP-TSN, the results were CER-NtFT4 = 3.8%, CER-CG7054 = 0.4% and CER = 0.0% (Figure 4B). There was no overlap between the interactions detected in the *in vivo* Y2H assay and those based on protein complexes extracted from Drosophila S2 cells.

According to Flybase and the String database [31, 32], the NtFT4 interaction partners in Drosophila include proteins associated with chaperone-mediated protein folding (CCT2, CCT7 and HSP26), protein ubiquitination (CG7220) and phosphorylation (RHEB and PEN), stress responses (CG7220, RHEB, HSP26 and TSN) and longevity (HSP26, RHEB and PyK) (Supplementary Figure 9). The results for RHEB and PyK revealed only indirect links to longevity via their interaction network (RHEB; Supplementary Figure 9) or an ortholog (Pyk in *Caenorhabditis elegans*) [33]. However, there is direct evidence that the small heat shock protein family is sufficient to promote longevity in flies [34]. Furthermore, the interaction between NtFT4 and HSP26 is highly conspicuous given the strength of the interaction suggested by FRET and co-immunoprecipitations experiments (Figure 4, Supplementary Figure 10). We therefore investigated the relationship between HSP26 and NtFT4 in more detail.

### NtFT4 interacts with HSP26 and stabilizes its expression in older flies

**Figure 5:**
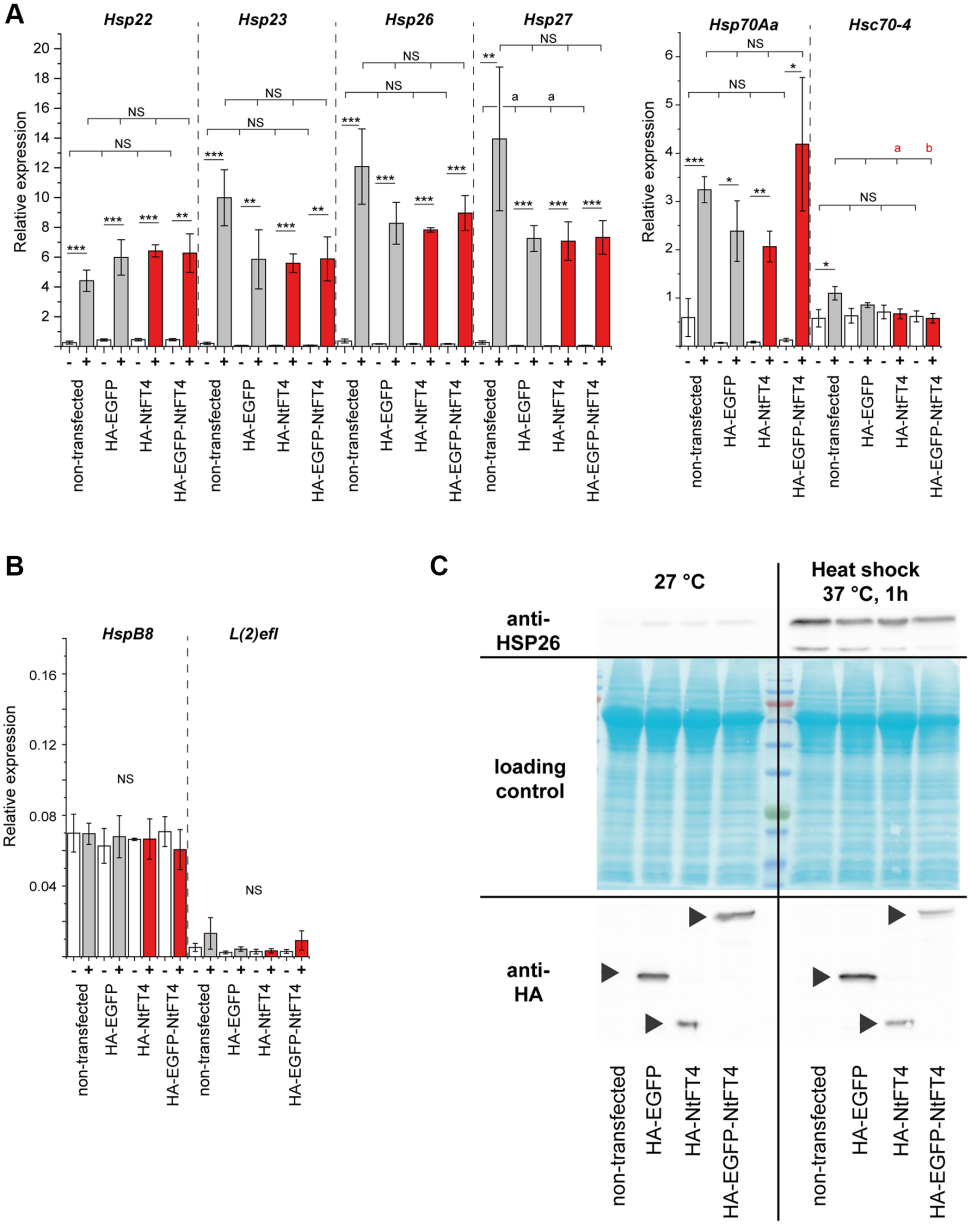
**Transfection and heat stress response of heat shock proteins in S2 cells.** Expression of stress-responsive (*Hsp22, Hsp23, Hsp26, Hsp27, Hsp70Aa* and *Hsc70–4*) (**A**) and non-responsive (*HspB8* and *l(2)efl*) (**B**) heat shock protein genes in S2 cells after transient transfection with HA-EGFP, HA-NtFT4 or HA-EGFP-NtFT4 compared to non-transfected cells. After transfection and induction of gene expression, cells were cultivated at 27°C (–, white bars) or stressed by heat shock at 37°C for 1 h (+, gray bars show controls and red bars show NtFT4). Relative gene expression was analyzed by qRT-PCR using *Gapdh2* as a reference. Data are means ± SEM (*n* = 3). Significance was tested by one-way ANOVA and Tukey’s *post hoc* test for responses to transfection (untransfected vs. HA-EGFP vs. HA-NtFT4 vs. HA-EGFP-NtFT4; *a* = significant compared to nontransfected cells, *p* < 0.1; *b* = significant compared to nontransfected cells, *p* < 0.05; Abbreviation: *NS*: not significant including all remaining comparisons) and using a *t*-test for pairwise comparisons of individual responses to heat shock (^****^p < 0.001, ^***^p < 0.01, ^**^p < 0.05, ^*^p < 0.1, Abbreviation: *NS*: not significant). (**C**) Immunodetection of HSP26 following the transient transfection of S2 cells with HA-EGFP, HA-NtFT4 or HA-EGFP-NtFT4 compared with nontransfected cells. The response of HSP26 to transfection and to heat shock at 37°C was analyzed 1 h after treatment by extracting proteins for immunodetection using anti-HSP26 antibodies (top right). The transient expression of HA-EGFP, HA-NtFT4 or HA-EGFP-NtFT4 was confirmed using anti-HA antibodies (bottom, arrowheads). All *p*-values are provided in Supplementary Table 9.

Heat shock proteins are often induced by stress, particularly heat stress. We observed no significant upregulation of any heat shock gene following transfection with *NtFT4* or any other construct (Figure 5A, 5B). However, we detected significant increases in the expression of *Hsp22*, *Hsp23*, *Hsp26*, *Hsp27* and *Hsp70Aa* under heat stress, regardless of transfection. We also confirmed the accumulation of HSP26 protein in response to heat stress but not transfection (Figure 5C). Neither transfection nor heat shock affected the expression of *HspB8*, *l(2)efl* or *Hsc70*–*4*. These data suggest that NtFT4 expression *per se* does not elicit a stress response.

**Figure 6:**
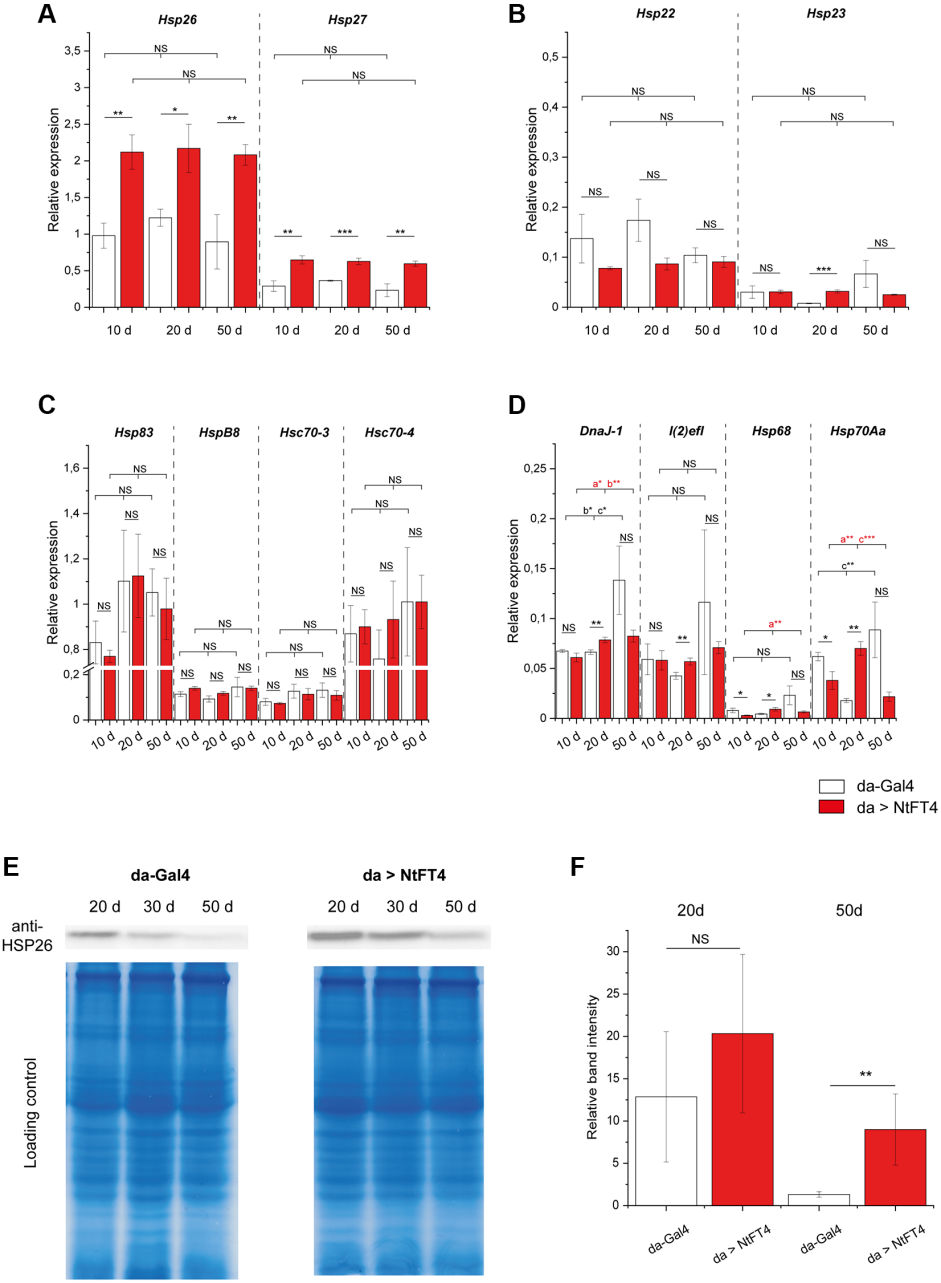
**Expression of heat shock genes during aging in flies expressing NtFT4.** Relative expression of two small heat shock protein genes directly associated with aging (*Hsp26* and *Hsp27*) (**A**), of the two small heat shock protein genes *Hsp22* and *Hsp23* (**B**), of larger heat shock protein genes *Hsp83*, *HspB8*, *Hsc70-3* and *Hsc70-4* (**C**), and of weakly-expressed heat shock protein genes *DnaJ-1*, *l(2)efl*, *Hsp68* and *Hsp70Aa* (**D**) in female *da > NtFT4* flies aged 10, 20 and 50 d, compared with *da-Gal4* flies by quantitative RT-PCR. Relative expression was calculated using *Gapdh2* as a reference gene. Data are means ± SEM (*n* = 3). Significance was tested by one-way ANOVA and Tukey’s *post hoc* test for changes during age (10 d vs. 20 d vs. 50 d) and using a *t*-test for pairwise comparisons between *da-Gal4* and *da > NtFT4* flies (^***^p < 0.01, ^**^p < 0.05, ^*^p < 0.1, Abbreviation: *NS*: not significant; *a* = significant between 10 d and 20 d, *b* = significant between 10 d and 50 d, *c* = significant compared between 20 d and 50 d). (**E**) Western blot showing the detection of HSP26 in protein extracts from female *da > NtFT4* flies aged 10, 20 and 50 d, compared with *da-Gal4* flies. A representative Western blot is shown for anti-HSP26 and comparable protein loading was ensured by staining with Coomassie Brilliant Blue. (**F**) Quantification of relative band intensities from three independent Western blot samples from 20 d (highest levels of HSP26 protein) and 50 d old flies. The relative band intensity was measured with imageJ and calculated by referring to the weakest band on each blot (50 d old *da-Gal4* flies). Data are means ± SEM (*n* = 3), *p* = 0.029 (*t*-test), Abbreviation: *NS*: not significant. The *p*-values of all comparisons are provided in Supplementary Table 9.

The expression of *Hsp26* and *Hsp27* decreases as flies age [35]. Accordingly, we quantified the expression of different heat shock family members at different ages in flies, revealing that NtFT4 significantly enhances the expression of *Hsp26* and *Hsp27,* which encode the most abundant members of the small heat shock protein family (Figure 6A–6D). In contrast, NtFT4 did not affect the expression of *Hsp83* and *Hsc70*–*4*, which encode the most abundant larger heat shock proteins (Figure 6C). No consistent correlation between NtFT4 and the expression of genes encoding other small (*Hsp22* and *Hsp23*) or larger (*HspB8, Hsc70*–*3, DnaJ-1, l(2)efl, Hsp68* and *Hsp70Aa*) heat shock proteins was observed, emphasizing the specific link between NtFT4 and *Hsp26* and *Hsp27*. The expression of *Hsp26* was mirrored by the abundance of HSP26 protein, which decreased stepwise in control flies aged 20+ days, eventually becoming barely detectable after 50 days (Figure 6E, 6F). The abundance of HSP26 also decreased with age in flies expressing NtFT4, but the rate of decline was shallower and the protein was still detectable in flies aged 50 d, comparable to the levels at 30 d in control flies (Figure 6E).

### NtFT4 induces differential gene expression related to metabolism and proteostasis

The nuclear localization of plant PEBPs in Drosophila cells suggests that their effect on longevity may reflect their ability to regulate transcription or mRNA metabolism. Genome-wide transcriptome analysis was therefore carried out to identify dysregulated genes using the Affymetrix GeneChip Drosophila Genome 2.0 array. We used female flies due to their pronounced longevity phenotype. Overall, we observed a high correlation in gene expression between control flies and those expressing NtFT4, as shown by correlation coefficients ranging from 0.973 to 0.997 (Supplementary Table 3). We detected 49 genes with significant upregulation and 100 with significant downregulation, defined as a fold change of at least 1.5 with a *p*-value less than 0.05 (Supplementary Table 4).

**Figure 7:**
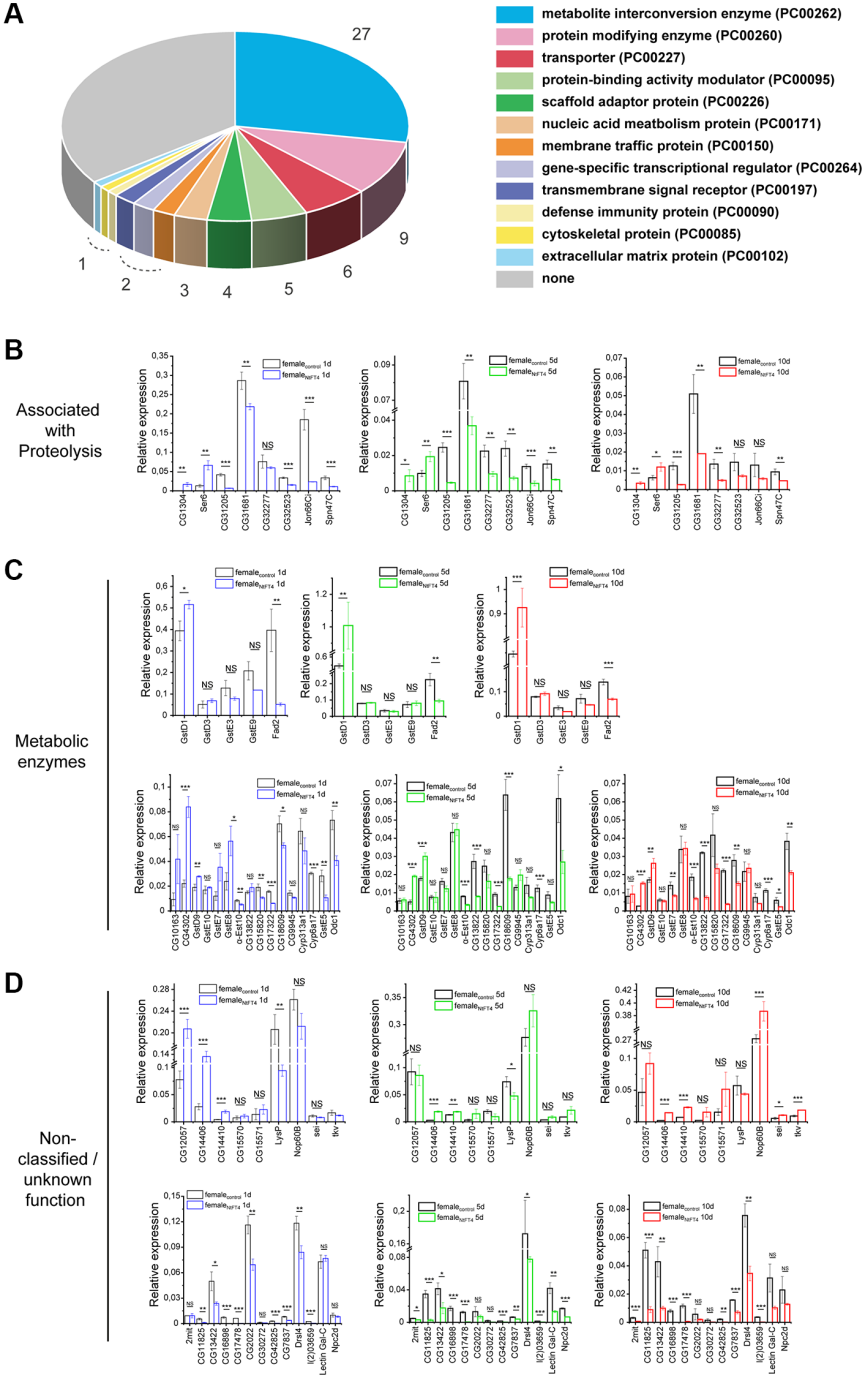
**GeneChip 2.0 array and gene expression analysis of female flies expressing NtFT4.** (**A**) Protein classes encoded by differentially expressed genes which were identified in the GeneChip Drosophila Genome 2.0 arrays (Affymetrix). We identified 149 genes that were significantly deregulated in female flies expressing NtFT4, 97 of which were mapped in the Panther database, and 63 genes were classified as representing 12 different protein classes. The largest protein classes were PC00262 (metabolite interconversion enzymes, 27 genes) and PC 00260 (protein modifying enzymes, 9 genes). Significance was determined using the paired *t*-test. Deregulated genes were included with a log_2_ fold change > 1.5 and a *p*-value < 0.05, *n* = 3. (**B**–**D**) Gene expression analysis. Deregulated genes associated with proteolysis (*CG1304*, *Ser6*, *CG31205*, *CG31681*, *CG32277*, *CG32523*, *Jon66Ci* and *Spn47C*) (**B**), annotated as *metabolic enzymes* (**C**), or genes which cannot be classified into groups and genes of unknown function (*non-classified/unknown function*) (**D**) identified by transcriptome analysis were analyzed individually in 1d (left, blue), 5 d (middle, green) or 10 d (right, red) old female flies expressing NtFT4 (*da > NtFT4*) compared with control (*da-Gal4*) flies (black). Relative expression levels were calculated in relation to the reference genes *Gapdh2*, *14-3-3 ε* and *RpL32*. Data are means ± SEM (*n* = 3), *p*-values are based on a *t*-test of pairwise comparisons between *da > NtFT4* and *da-Gal4* flies, ^****^p < 0.001, ^***^p < 0.01, ^**^p < 0.05, ^*^p < 0.1, Abbreviation: *NS*: not significant. The *p*-values of all comparisons are provided in Supplementary Table 9.

The low number of modulated genes facilitated the subsequent verification of differentially expressed genes as well as functional enrichment analysis. The expression of NtFT4 mainly affected genes involved in metabolic processes (Supplementary Tables 5 and 6), specifically 27.8% of the modulated genes were assigned to the protein class *metabolite interconversion enzyme* and 9.3% to the class *protein modifying enzyme* (Figure 7A, Supplementary Table 6). In the latter, eight of the nine identified gene products were annotated as proteases and four others (Jon66Ci, CG31205, CG11841 and CG42694) were putative proteases containing peptidase sequence motifs (Table 2). We also found four uncharacterized proteins that may function as protease inhibitors or regulators of proteolysis (the serpins Spn47C and Spn43Ab, and the Kazal-domain proteins Kaz1-ORFB and CG1077).

**Table 2:** Genes encoding proteases or their inhibitors that are deregulated in female flies expressing *NtFT4*.

**UniProtKB**	**Mapped IDs**	**Gene group (Flybase)**	**Symbol**	**FC**	***p*-value**
Q8IN51	NM_169915	S1A non-peptidase homolog	*CG31205*	−4.27	1.33 × 10^−2^
Q9VSJ1	NM_168271	S1A Serine proteases - Elastase-like	*Jon66Ci*	−3.90	9.50 × 10^−4^
Q8IPY7	NM_164473	S1A Serine proteases - Trypsin-like	*CG31681*	−2.26	4.05 × 10^−3^
Q8IQ51	NM_167738	S1A Serine proteases - Trypsin-like	*CG32523*	−2.26	1.16 × 10^−2^
Q8IRE1	NM_168002	S1A Serine proteases - Trypsin-like	*CG32277*	−2.03	4.72 × 10^−2^
Q9VAQ4	NM_143404	S1A Serine proteases - Trypsin-like	*CG11841*	−1.93	3.48 × 10^−2^
Q9VQA0	NM_134818	S1A Serine proteases - Trypsin-like	*Send1*	−1.67	3.72 × 10^−2^
Q9VAS2	NM_143386	Neprilysin-like metalloendopeptidase	*CG14528*	−1.63	2.77 × 10^−2^
A0A0B4JD89	NM_001202065	S1A non-peptidase homolog	*CG42694*	−1.61	1.79 × 10^−2^
Q9VRD1	NM_134574	S1A Serine proteases - Elastase-like	*CG1304*	3.02	9.10 × 10^−3^
Q9VRD0	NM_078702	S1A Serine proteases - Elastase-like	*Ser6*	2.00	4.28 × 10^−2^
Q9VMM2	NM_135117	Dipeptidyl peptidases IV	*CG11034*	1.97	2.07 × 10^−2^
Q7K508	NM_001169645	Putative non-inhibitory serpin	*Spn47C*	−2.10	1.42 × 10^−2^
A1Z6V5	NM_001032224	Putative non-inhibitory serpin	*Spn43Ab*	−1.66	1.85 × 10^−2^
Q9VNL6	NM_141360	Kazal domain superfamily	*CG1077*	4.70	2.79 × 10^−2^
O97042	NM_001031920	Kazal domain superfamily	*Kaz1-ORFB*	−1.55	2.62 × 10^−2^

GeneChip Drosophila Genome 2.0 arrays (Affymetrix) revealed several deregulated proteases, protease inhibitors and uncharacterized proteins associated with proteolysis or its regulation [36, 37]. Fold-changes were calculated in comparison with *da-Gal4* flies and *p*-values were calculated using parametric *t*-tests.

Quantitative RT-PCR analysis confirmed the differential expression of genes encoding the predicted proteases CG1304, CG31205, CG31681, CG32277, CG32523, Jon66Ci and Ser6, and the serpin Spn47C (Figure 7B). We also confirmed the differential expression of genes involved in metabolic processes. The significantly downregulated genes in flies expressing NtFT4 included *Cyp6a17*, *Fad2*, *CG17322*, *CG18609*, *α-Est10* and *GstE5*, whereas *CG15661*, *CG4302*, *CG15334*, *CG7900*, *GstD1* and *GstD5* were significantly upregulated (Figure 7C). We also confirmed the differential expression of *CG14406*, *CG14410*, *CG15570*, *CG12057*, *sei* and *tkv* (upregulated), as well as *CG16898*, *CG17478*, *l(2)03659*, *CG42825*, *CG11825*, *CG30272* and *CG13422* (downregulated), which either await functional characterization or cannot be grouped according to their functions (Figure 7D).

We also looked at direct molecular markers of aging. Protein carbonylation results from oxidative damage that accumulates with age. Given the numerous differentially expressed genes and interacting proteins involved in proteostasis, we also tested whether long-lived flies expressing NtFT4 or CG7054 had lower protein carbonylation levels. However, there were no significant differences in protein carbonylation when comparing either *NtFT4* or *CG7054* expressing flies to controls at 10 or 30 days old (Supplementary Figure 11).

## DISCUSSION

We investigated the activity of animal PEBPs expressed in Arabidopsis and tobacco, and of tobacco PEBPs expressed in Drosophila and human cells, as well as transgenic flies. The heterologous expression of the plant PEBPs (NtFT4 and NtFT2) in Drosophila resulted in a significant increase in longevity. In contrast, the expression of several animal PEBPs in plants had no significant effect on growth or development, including the floral transition. Although the animal PEBPs interacted with canonical partners of FT-like proteins in plants, the interactions with NtFD1 and 14-3-3 proteins were not sufficient to overcome endogenous regulatory cues controlling developmental transition. The nonreciprocal activity of plant and animal PEBPs may reflect differences in protein stability, subcellular localization or interaction partners [27].

Drosophila PEBPs are structurally similar to human PEBP1 (RKIP) and the crystal structure of CG7054 has been solved [38]. The structures share a short helical region at the C-terminus which is entirely missing from all plant PEBPs (Supplementary Figure 4). Instead, the C-terminal region of plant FT-like proteins features a protease cleavage site, which allows posttranslational modification [39]. The presence of this cleavage site could reduce the stability of heterologous plant PEBPs in animal cells and may contribute to the low NtFT protein levels we detected.

The known functions of PEBPs include the regulation of developmental transitions in plants and the regulation of cell survival, proliferation and differentiation in mammals [1–3, 17, 18, 20, 40]. The heterologous expression of NtFT4 in flies revealed new aspects of PEBP activity that point to a role in proteostasis, improving health and lifespan [41]. Mammalian and Drosophila PEBPs can interfere with protein kinase activity [4, 12, 42]. In humans, the inhibition of kinase signaling by RKIP depends on phosphorylation, which facilitates interactions with target kinases [1, 43–45].

Drosophila PEBPs are associated with fitness through their role in innate immunity, which is evidenced by the upregulation of PEBP genes during infections [13, 14] and the protection against bacterial infections conferred by the overexpression of *PEBP1* [16]. Our data provide additional links between PEBPs and fitness by demonstrating their impact on longevity and motility. First, we found that the ubiquitous knockdown of *CG7054* expression causes late pupal lethality in ~40% of the animals. Similarly, the knockdown of *CG7054* or *a5* was shown to be partly lethal in genome-wide RNAi experiments [29, 46]. As part of a systemic approach to assess muscle morphogenesis, the lethal effect of *CG7054* knockdown has been demonstrated at the late pupal stage when using the muscle-specific driver *Mef2-Gal4* [29, 47]. In addition to partial lethality, we demonstrated that the surviving adult flies showed reduced locomotor activity and the adult lifespan was significantly shorter. This complements our finding that the overexpression of either *CG7054* or *NtFT4* increases the longevity of flies. The expression of *NtFT4* not only increases the lifespan of flies but also counteracts age-related deterioration in locomotor behavior, one of the most serious behavioral disorders in old age [48]. Here we noted an interesting sex difference. Whereas *NtFT4* expression did not improve the locomotor abilities of young females, there were significant benefits in males and older females. There appears to be a maximum level of activity that cannot be improved in young female flies. In contrast, young males are generally less motile than young females but their locomotor activity is significantly enhanced by PEBP expression.

Some components of the NtFT4 interactome in Drosophila are already known to be associated with longevity, including PyK, RHEB and HSP26 [33, 34, 49, 50–53]. PyK and RHEB regulate mTOR kinase activity [49, 54, 55], thus the interaction with NtFT4 resembles the canonical regulatory mechanism of PEBPs. A link with the insulin/IGF and TOR signaling pathway (IIS/TOR), which also connects metabolism with cellular homeostasis and aging [56–58], is also supported by the interaction between NtFT4 and CCT7 (or other chaperonin-containing TCP1 subunits) [55]. CCTs are also targets of phosphorylation by RSK or S6K, downstream of mTOR activation by insulin [59]. The interactions with PyK, RHEB and CCTs may therefore integrate NtFT4 into the signaling network that controls longevity (Supplementary Figure 12).

The interaction between NtFT4 and HSP26 reveals a new mechanism of PEBP activity. Heat shock proteins are generally associated with cellular stress responses and their role is to protect cells from the effects of damaged and misfolded proteins [60–64]. If such proteins persist in the cytoplasm, three chaperone-mediated quality control pathways can be induced: partly denatured proteins can be recognized by heat shock proteins and refolded to retain their function, whereas damaged proteins can be cleared by HSP70-dependent degradation via the proteasome or by chaperone-mediated autophagy [65, 66]. NtFT4 appears to integrate with this system by stabilizing HSP26 levels, which in turn promotes general protein refolding. Moreover, the proteases deregulated by NtFT4 expression in flies may contribute to protein degradation during autophagy. Interestingly, phosphatidylethanolamine (one of the phospholipid ligands of PEBPs) has been shown to induce autophagy, extend the lifespan of *Saccharomyces cerevisiae* [67], and also act as a chaperone for membrane proteins [68, 69].

The small heat shock proteins of Drosophila show functional diversity, with some facilitating protein refolding and others preventing the accumulation of toxic proteins. Regardless of their task in the proteome maintenance system, the overexpression of these diverse small heat shock proteins increased the longevity of fruit flies [34, 70, 71]. Interestingly, NtFT4 not only interacts physically with HSP26 but also upregulates *Hsp26* gene expression. The conspicuous nuclear localization of NtFT4 supports the hypothesis that NtFT4 not only interacts with the cytoplasmic proteostasis machinery but also participates in the transcriptional regulation of its components, which are needed to maintain cell integrity (Supplementary Figure 12). Many proteins found in nuclear speckles, where NtFT4 was enriched, are involved in the regulation of transcription and RNA splicing [72, 73].

In summary, we identified a novel mechanism that connects PEPBs to aging. We found that a plant PEBP (NtFT4) increases longevity in Drosophila by interacting with a number of proteins involved in proteostasis, including HSP26. The functional specificity of different members of the PEBP family highlights their complex molecular interactions, but also provides many opportunities to modulate their activity. NtFT4 also provides a powerful tool to investigate the regulation of proteostasis in animals.

## MATERIALS AND METHODS

### Reagents, plasmids and cloning

All primers used for cloning are listed in Supplementary Table 7. Accession numbers for genes and proteins are listed in Supplementary Table 8. Cloning steps are described in more detail in the Supplementary Methods.

For Drosophila transformation, the NtFT2, NtFT4 and CG7054 coding sequences were amplified by PCR using primers with attached restriction sites, and were transferred to pENTR4 vectors by restriction and ligation. Subsequent transfer to vector pUASTattB_rfA or pUASTattB_rfA_3xHA [28] was achieved by Gateway recombination. Cloning steps for plasmids used in the transfection of yeast, *N. benthamiana* epidermal cells, HEK-293T and S2 cells are provided in the Supplementary Methods.

### Plant cultivation and transformation

Tobacco (*Nicotiana tabacum* cv. SR1) and Arabidopsis (*Arabidopsis thaliana* ecotype Col-0) plants were cultivated and transformed using the leaf disc method (tobacco) or by floral dip (Arabidopsis) as previously described [18]. Cultivation and transformation details are provided in the Supplementary Methods.

### Bimolecular fluorescence complementation

Transient expression of split-mRFP and Venus fusion constructs in *N. benthamiana* plants was carried out as previously described [18]. More details are provided in the Supplementary Methods. Fluorescence was analyzed using a TCS SP5 X confocal laser scanning microscope (Leica Microsystems) at excitation/emission wavelengths of 514/525–600 nm for Venus and 543/569–629 nm for reconstituted mRFP. All combinations of split mRFP constructs (C-terminal or N-terminal fusion to CmRFP and NmRFP) were tested. Interaction was confirmed if at least five independent images showing fluorescence were captured.

### Drosophila work

Flies were raised at 25°C and transgenes were introduced by φC31-based transformation at the landing site *86Fb* [28]. Gain-of-function studies were carried out using the Gal4/UAS system [74]. The driver *da-Gal4* was obtained from the Bloomington stock center. *CG7054* was knocked down using the dsRNA-GD12116 strain obtained from the Vienna stock center (VDRC #40415).

### RING assay

Negative geotaxis was monitored as previously described [30]. At least 100 male and female flies (10, 30 or 45 days old) were collected in groups of 10 in fresh vials with standard food. After a recovery period of 24 h, they were transferred to test tubes without anesthesia. After 5–10 min to acclimate to the new environment, the tubes were tapped five times in a custom-made device to ensure consistent forces [30]. After impact, the position of each fly within the tube was recorded for 10 s at 10 frames/s. After a 2-min rest period, the tapping process was repeated and the same flies were observed again, for a total of five tests. Images were processed using Fiji with the MTrack3_.jar plugin and AutoRun2.ijm macro. The mean velocity was determined using RING assay Script.R in the R program.

### Immunofluorescence staining of larval tissue

Tissues were fixed and prepared for immunofluorescence as previously described [75]. The HA-tagged NtFT4 and NtFT2 proteins were detected using a mouse anti-HA antibody (Covance) and anti-mouse IgG coupled to Alexa 488, 568 or 647 (Thermo Fisher Scientific). Nuclei were counterstained with 4′,6-diamidino-2-phenylindole (DAPI). Specimens were analyzed using a Zeiss LSM710 or LSM880 confocal microscope. Original confocal data were processed using ZEN 2012 software (Zeiss), Adobe Photoshop CS6, and Fiji [76].

### Cell culture and transfection

S2R+ cells (Drosophila Genomics Resource Center, NIH Grant 2P40OD010949) are described herein as S2 cells. The cells were cultivated at 27°C in Schneider’s Drosophila medium with 5% fetal calf serum and a 1% antibiotic-antimycotic mix (all from Thermo Fisher Scientific) in six-well plates for transfection and in T25 flasks for subculturing. HEK-293T cells were grown in RPMI-1640 GlutaMAX medium with 5% fetal calf serum and a 1% antibiotic-antimycotic mix in a 5% CO_2_ atmosphere at 37°C with a relative humidity of ~93%. Cells were transferred to six-well plates in Opti-MEM for transfections using Lipofectamine 3000 (Thermo Fisher Scientific) according to the manufacturer’s protocol. To induce expression of constructs using the pMT plasmids, S2 cells were treated with 5 mM CuSO_4_.

### Protein extraction, analysis and Western blotting

Proteins for direct immunodetection were extracted from snap-frozen flies, S2 or HEK293T cells using a Tris lysis buffer (Tris-HCl pH 7.5, 150 mM NaCl, 1 mM EDTA, 1% (v/v) NP-40 containing protease and phosphatase inhibitor cocktails). Proteins were separated by SDS-PAGE and transferred to a 0.2-μm nitrocellulose membrane using the wet Mini Trans-Blot Cell system (Bio-Rad Laboratories). Western blots were probed with the following antibodies: anti-HA tag rabbit polyclonal (MBL; #561), anti-Myc tag mouse monoclonal (MBL; #047-3), and anti-HSP26 rabbit polyclonal (custom made, Proteogenix). The primary antibodies were detected using either anti-rabbit/anti-mouse IgG secondary antibodies coupled to alkaline phosphatase (Thermo Fisher Scientific) and SigmaFast BCIP/NBT tablets (Sigma-Aldrich), or anti-rabbit/anti-mouse IgG secondary antibodies coupled to horseradish peroxidase (Thermo Fisher Scientific) and the SuperSignal West dura kit (Thermo Fisher Scientific). More details are provided in the Supplementary Methods.

### Protein carbonylation analysis

Whole protein extracts were prepared from adult flies (10 or 30 days old). The total soluble protein concentration was measured using the RotiQuant Universal Kit (Roth), and 2–10 mg of protein was immediately used to measure carbonylation using the Protein Carbonyl Content Assay Kit (Sigma-Aldrich). Protein carbonylation was quantified by normalizing the value against the total protein concentration.

### LC-MS analysis

For immunoprecipitation, HA-tagged proteins were extracted from the cytosolic and nuclear fractions of transfected S2 cells using hypotonic and hypertonic extraction buffers. Both fractions were combined for subsequent immunoprecipitation using the Pierce Magnetic HA-Tag IP/Co-IP Kit (Thermo Fisher Scientific) according to the manufacturer’s acidic elution protocol. Eluates were analyzed by SDS-PAGE and silver staining using Pierce Silver Stain for Mass Spectrometry (Thermo Fisher Scientific) and interacting proteins were identified by LC-MS/MS from the whole eluates and from excised gel bands. Briefly, proteins from the eluates and from gel bands were digested with trypsin [77, 78], acidified with 1% (v/v) trifluoroacetic acid (TFA), desalted [79] and dried in a vacuum centrifuge for storage at –80°C. LC-MS/MS analysis was carried out with reconstituted peptides (2% (v/v) acetonitrile/0.05% (v/v) TFA) using an Ultimate 3000 nanoLC (Thermo Fisher Scientific) coupled via a nanospray interface to a Q Exactive Plus mass spectrometer (Thermo Fisher Scientific). Sample preparation and LC-MS/MS details are provided in the Supplementary Methods.

### Quantitative PCR

RNA was extracted from flies using the Quick-RNA Tissue/Insect Microprep kit (Zymo Research) and from cells using the NucleoSpin RNA kit (Macherey-Nagel) according to the manufacturers’ specifications. Following reverse transcription using PrimeScript RT master mix (Takara Bio), gene expression was analyzed by quantitative real-time PCR using Kapa SYBR Fast qPCR Master Mix and the CFX96 Real-Time System (Bio-Rad Laboratories). Each reaction was carried out in technical triplicates and the primer sequences are provided in Supplementary Table 7. Specificity was ensured by melt curve analysis and the sequencing of PCR products, and by including no-template and no-reverse-transcription controls. Individual PCR efficiency was determined using LinReg PCR v2017.0 [80] and relative gene expression levels were normalized to *Gapdh2* (S2 cells) or to the mean of *Gapdh2*, *14-3-3ε* and *RpL32* (flies).

### Live-cell imaging for subcellular localization

Localization studies using the pcDNA3 vectors containing constructs HA-EGFP-NtFT4, HA-EGFP-CG7054, HA-EGFP-PEBP1, HA-EGFP-10298, HA-EGFP-CG6180, HA-EGFP-CG17917, HA-EGFP-CG17979 and Myc-mRFP-H2AZ were carried out by co-transfecting HEK-293T cells with EGFP plasmids and pcDNA3-Myc-mRFP-H2AZ using Lipofectamine 3000. Cells in six-well plates were transiently transfected in Opti-MEM medium and fluorescence was imaged in living cells 24 h post-transfection using a TCS SP5 X confocal scanning laser microscope.

### GeneChip analysis

RNA was extracted from female flies (*da > NtFT4* and *da-Gal4* as a control) at ages of 0–24 h (described herein as 1 day), 5–6 days (5 days) or 10–11 days (10 days) using the Quick-RNA Tissue/Insect Microprep kit, and equimolar amounts representing each age were pooled. Affymetrix GeneChip Drosophila Genome 2.0 Array analysis was carried out by IMGM Laboratories. More details are provided in the Supplementary Methods. For the identification of genes with significant differences in expression in pairwise comparisons, different filtering approaches were tested using both the FDR-corrected *p*-value (Benjamini-Hochberg) and the non-corrected *p*-value from the paired *t*-test. Sequences for subsequent verification of differential gene expression were retrieved from Flybase FB2021_02 [31].

### Yeast-two hybrid screening and drop test

The initial Y2H screen was carried out using the Matchmaker GoldYeast Two-Hybrid System (Takara Bio), the Mate and Plate Library - *Universal Drosophila (Normalized)* (Takara Bio) and pGBKT7-NtFT2 as a bait construct introduced into *S. cerevisiae* strain Y2HGold using the Yeastmaker transformation system 2 (Takara Bio). To confirm interactions, full-length coding sequences were introduced into pGADT7 and introduced into *S. cerevisiae* Y2HGold cells along with pGBKT7 and applied to drop tests. Co-transformation of pGBKT7-53 and pGADT7-T served as a positive control, and co-transformation of pGBKT7-Lam and pGADT7-T served as a negative control (Takara Bio). Further details are provided in the Supplementary Methods.

### FRET analysis

The *NtFT4* and *CG7054* coding sequences were cloned in-frame with mCerulean (Cer), whereas *CCT7, CG4364, Df31, Hsp26, p47, Pen, Pyk* and *Tsn* were cloned in-frame with mEYFP (EYFP) in vector pcDNA3, with the fluorescent proteins separated from their fusion partners by the linker sequence (GGGGS)_3_. A fusion of Cer and EYFP in pcDNA3 was prepared as a positive control, whereas Cer or EYFP (each fused only to the linker sequence) were prepared as negative controls. HEK-293T cells were transfected with appropriate combinations of plasmids using Lipofectamine 3000, and FRET was analyzed 24 h post-transfection by flow cytometry using a BD FACSCelesta with BVYG laser configuration (BD Biosciences). The gating strategy and controls are provided in the Supplementary Methods.

### Identification of interaction networks

To integrate NtFT4 into functional networks, its interaction partners were analyzed using Flybase FB2021_02 [31] to identify functional overlaps and they were used for single protein analysis in the String database (https://string-db.org) [32]. Here, interaction sources were set to include interactions based on text mining, experimental evidence, databases, co-expression, neighborhood, gene fusion or co-occurrence.

### Statistical analysis

All boxplots in the figures were prepared in OriginPro2020 v9.7.5.184 (OriginLab) using the default settings (center line = median; box limits = upper and lower quartiles; whiskers = 1.5× interquartile range; points = outliers). Statistical analysis, if not stated otherwise, was carried out using OriginPro2020. Differences in lifespan were analyzed using Kaplan-Meier survival curves and the Mantel-Cox (log-rank) test. Equality of variances was determined by one-way analysis of variance (ANOVA), and pairwise comparisons were assessed using Tukey’s *post hoc* test for multiple comparisons and Student’s *t*-test for single pairwise comparisons. All *p*-values that could not be provided in figure legends due to space constraints are summarized in Supplementary Table 9.

### Data availability

All data are available upon request. GeneChip data have been deposited in the ArrayExpress database at EMBL-EBI [81] (https://www.ebi.ac.uk/arrayexpress/experiments/E-MTAB-10730/).

## Supplementary Materials

Supplementary Methods

Supplementary Figures

Supplementary Tables 1-4

Supplementary Table 5

Supplementary Table 6

Supplementary Table 7

Supplementary Table 8

Supplementary Table 9
