# Supplementary material for: The tobacco phosphatidylethanolamine-binding protein NtFT4 increases the lifespan of Drosophila melanogaster by interacting with the proteostasis network
Source: Aging (Albany NY). 2022 Apr 8;14(7):2989–3029. doi: 10.18632/aging.204005 (PMC9037272; doi:10.18632/aging.204005)
Supplement: Supplementary Methods [file aging-14-204005-s001.pdf]

SUPPLEMENTARY METHODS

Cloning

To create stable plant lines expressing different PEBPs, we used the binary plasmids pLab12.1 [1] for Arabidopsis and pBIN19 [2] for tobacco. The coding sequences of human RKIP and PEBP4 (also known as hPEBP4), tobacco NtFT2 and NtFT4, and Drosophila PEBP1 and CG7054 were amplified by PCR using primers with attached restriction sites (corresponding restriction sites are noted in the primer names, and all restriction enzymes were from New England Biolabs), and were transferred to vectors pLab12.1 or pRT104 [3] by restriction and ligation. A variant of CG7054 containing part of NtFT4 (including the YAPGW and EVYN motifs in segment B) was prepared by splice overlap extension PCR and subsequent cloning as described for the other PEBPs (Supplementary Figure 1). The expression cassettes were transferred to pRT104, released with HindIII, and ligated into the final destination vector pBIN19.

For Drosophila transformation, the NtFT2, NtFT4 and CG7054 coding sequences were amplified by PCR using primers with attached restriction sites, and were transferred to pENTR4 vectors (Thermo Fisher Scientific) by restriction and ligation. Subsequent transfer to vector pUASTattB_rfA or pUASTattB_rfA_3xHA [4] was achieved by Gateway recombination.

For Y2H and BiFC assays, the coding sequences of NtFT2, NtFT4, NtFD1, 14-3-3 a-1, 14-3-3 c, 14-3-3 d, 14-3-3 e-2, 14-3-3 f, 14-3-3 f-1, 14-3-3 g and 14-3-3 i-2 from tobacco as well as 4E-T, Act42A, Cals, CCT7, CG3303, CG4364, CG5028, CG6523, CG7054, CG7220, CG11148, CG13775, CG31644, CKIIα-i3, Df31, DhpD, Dpr7, Eip55E, Hsp26, Idgf3, mRpL44, Nplp4, Nrv2, Nrv3, p47, Pen, PyK, Rheb, Rps10b, Tsn, Wech, Yippee and ε-Try from Drosophila were amplified from cDNA using primers with attached restriction sites, and transferred to vectors pGBKT7 or pGADT7 (Takara) or to pENTR4. Subsequent transfer to pBatTL vectors was achieved by Gateway recombination (BatTL plasmids were kindly provided by Joachim Uhrig and Guido Jach, University of Cologne, Cologne, Germany).

For transient expression in HEK-293T or S2 cells, the codon-optimized NtFT4 and NtFT2 coding sequences were synthesized as Gene Strings by Thermo Fisher Scientific, cloned in-frame with HA-EGFP, and transferred to vector pMT-puro (a gift from David Sabatini, Addgene plasmid # 17923; http://n2t.net/addgene:17923; RRID:Addgene_17923) or pcDNA3 (pcDNA3-EGFP was a gift from Doug Golenbock, Addgene plasmid # 13031; http://n2t.net/addgene:13031; RRID:Addgene_13031) by amplifying each segment, digesting the products with restriction enzymes SpeI/XhoI (HA-EGFP), XhoI/ApaI (NtFT4 or NtFT2) and SpeI/ApaI (pMT or pcDNA3 backbone) and ligating them. Accordingly, the Drosophila PEBPs CG7054 and PEBP1 and the putative interaction partners (14-3-3 ζ, Cbs, CCT2, CCT7, CG4364, Df31, HSP26, p47, Pen, PyK, Rack1, Tsn) for co-immunoprecipitation were amplified from cDNA and transferred by restriction and ligation into pMT-puro or pcDNA3. HA without EGFP and Myc tags was added to the coding sequences by PCR.

Plant cultivation and transformation

Tobacco (*Nicotiana tabacum* cv. SR1) seeds were sown and the plants were cultivated in soil under long-day conditions in the greenhouse (16-h photoperiod, artificial light switched on if natural light fell below 700 µmol m^{-2} s^{-1}, 22–25°C under light, 19–25°C in the dark). Stable transformation was carried out using the leaf disc method [5] with *Agrobacterium tumefaciens* strain LBA4404 [6]. For the selection of transgenic plants, MS medium was supplemented with 100 mg/L kanamycin. After callus regeneration and rooting in sterile culture medium, independent transgenic plant lines were cultivated in the greenhouse as stated above.

Arabidopsis (*Arabidopsis thaliana* ecotype Col-0) plants were cultivated in a York phytochamber at 23°C with a 16-h photoperiod (20 klx light intensity). Transformation was carried out by floral dip using *A. tumefaciens* EHA105 carrying the appropriate binary plasmids [7]. For the selection of transgenic plants, seeds were sown and seedlings were sprayed 2–3 times with glufosinate ammonium (trade name Basta).

Bimolecular fluorescence complementation

For the transient expression of split-mRFP and Venus fusion constructs, *A. tumefaciens* strain GV3101 pMP90 was transformed with the corresponding binary pBatTL destination vector by electroporation. *N. benthamiana* plants were cultivated in the greenhouse (16-h photoperiod) until they were 3–4 weeks old before infiltrating the leaves with *A. tumefaciens* strain GV3101 pMP90 carrying the appropriate pBatTL plasmids and *A. tumefaciens* strain C58C1 carrying the pCH32 helper plasmid and the pBin61 plasmid encoding the RNA silencing suppressor p19 from tomato bushy stunt virus [8]. Plants were cultivated under continuous light for 3–4 days, and leaf discs were screened for fluorescent cells in the abaxial epidermis.

Yeast-two hybrid screening and drop test

The initial Y2H screen was carried out using the Matchmaker GoldYeast Two-Hybrid System (Takara Bio), the Mate and Plate Library - *Universal Drosophila (Normalized)* (Takara Bio) and pGBKT7-NtFT2 as a bait construct introduced into *S. cerevisiae* strain Y2HGold according to the manufacturer's protocol (Takara Bio). Plasmids were isolated from positive colonies using the Zymoprep Yeast Plasmid Miniprep I kit (Zymo Research) for sequencing. To confirm interactions, full-length coding sequences were introduced into pGADT7 and introduced into *S. cerevisiae* Y2HGold cells along with pGBKT7. Transformed colonies were selected by growth on double dropout (DDO) medium plates (SD –leucine –tryptophan) (Takara Bio). For drop tests, yeast strains were grown in 3 mL DDO liquid medium at 30°C until they reached OD_{600} = 1, then 10 µL of the undiluted culture and 1:10, 1:100 and 1:1000 dilutions) was dropped onto selective quadruple dropout medium (SD –leucine –tryptophan –adenine –histidine) containing 200 ng/mL aureobasidin A (Takara Bio) and incubated at 30°C until colony growth was clearly observed for the positive control.

GeneChip analysis

After measuring the RNA concentration and purity on a NanoDrop ND-1000 spectral photometer (Peqlab), RNA integrity was confirmed by capillary electrophoresis using a 2100 Bioanalyzer and the RNA 6000 Nano LabChip Kit (Agilent Technologies). We introduced 200 ng total RNA per sample into an RT-IVT reaction after spiking the RNA samples with polyadenylated transcripts using the Gene Chip Poly-A Control kit (Affymetrix) serving as an internal labeling control for linearity, sensitivity and accuracy.

The spiked total RNA was reverse transcribed into cDNA and then converted into biotin-labeled antisense RNA by 16-h *in vitro* transcription using the 3′IVT Expression kit (Affymetrix). The resulting single-stranded antisense RNA was purified and fragmented. Following the validation of antisense RNA quality, the labeled and fragmented RNA was spiked with cDNA hybridization controls (GeneChip Hybridization Control Kit, Affymetrix). The spiked RNA samples were hybridized at 45°C for 16 h on separate Affymetrix GeneChip Drosophila Genome 2.0 Arrays.
After hybridization, microarrays were stained in two binding cycles using anti-biotin antibodies and streptavidin, R-phycoerythrin conjugate. The microarrays were then washed with increasing stringency and conserved in holding buffer using the Affymetrix GeneChip 3000 Fluidics Station in combination with the Affymetrix GeneChip Command Console (AGCC) – Fluidics Control Software v4.0.0.1567. Fluorescence was detected using the Affymetrix GeneChip 3000 Scanner and AGCC Scan Control Software v4.0.0.1567 (Affymetrix). The software tool GeneSpring GX13.1 (Agilent Technologies) was used for quality control, statistical data analysis, visualization and differential expression analysis. The Robust Multi-Array Analysis (RMA) algorithm was applied for summarization and quantile normalization of the dataset. Pearson's correlation coefficients (r) were calculated for all pairwise comparisons.

Protein extraction, analysis and western blotting

For protein extraction and direct immunodetection, snap-frozen flies were homogenized in ice-cold lysis buffer (Tris-HCl pH 7.5, 150 mM NaCl, 1 mM EDTA, 1% (v/v) NP-40 containing protease and phosphatase inhibitor cocktails) using a micro-pistil, and S2 or HEK-293T cell pellets were lysed without homogenization. For the isolation of total proteins to test protein carbonylation, snap-frozen flies were homogenized in ice-cold lysis buffer 2 (20 mM HEPES pH 7.9, 420 mM NaCl, 1.5 mM $MgCl_2$, 0.2 mM EDTA, 1 mM DTT, 25 % (v/v) glycerol containing protease and phosphatase inhibitor cocktails) using a micro-pistil, followed by two freeze-thaw cycles (–20/95°C) and three rounds of sonication for 30 s in a water bath. After these homogenization procedures, proteins were extracted on ice for 30 min and debris was removed by centrifugation (20,000 × g, 20 min, 4°C). Protein concentrations in the extracts were measured using the Pierce Coomassie Plus Protein Assay (Thermo Fisher Scientific) or the RotiQuant Universal assay (Roth) according to the manufacturers' recommendations. Proteins were separated by SDS-PAGE and stained using the PAGE Blue protein staining kit or transferred to a 0.2-µm nitrocellulose membrane using the wet Mini Trans-Blot Cell system (Bio-Rad Laboratories). Transfer and comparable protein loading were controlled by staining blots with Ponceau S or the Pierce Reversible Protein Stain Kit for Nitrocellulose Membranes (Thermo Fisher Scientific). Anti-HSP26 rabbit polyclonal antibodies were custom made using three peptides (VDELQEPRSPIYEL, LPLGTQQRRSINGC and VLALRREMANRND) for immunization (Proteogenix). All primary antibodies were detected using either anti-rabbit/anti-mouse IgG secondary antibodies coupled to AP (Thermo Fisher Scientific) and SigmaFast BCIP/NBT tablets (Sigma-Aldrich), or anti-rabbit/anti-mouse IgG secondary antibodies coupled to HRP (Thermo Fisher Scientific) and the SuperSignal West dura kit (Thermo Fisher Scientific).

The signals from the SuperSignal West dura kit were detected using a G:Box Chemi (Syngene). Brightness and contrast were optimized using Adobe Photoshop CS6 v13.0.1 × 64 (Adobe Systems).

Protein extraction and LC-MS analysis

Transiently transfected cells were harvested by aspiration, washed with cold PBS and proteins were extracted in two steps under mild conditions to maintain interaction complexes. First, cells were resuspended in a hypotonic buffer to extract cytoplasmic proteins (10 mM HEPES pH 7.9, 10 mM KCl, 1.5 mM $MgCl_2$, 1 mM DTT containing protease and phosphatase inhibitor cocktails) for 30 min on ice. Cytoplasmic proteins were collected in the supernatant by centrifugation (4000 × g, 10 min, 4°C) and the nuclear pellet fraction was resuspended in extraction buffer (20 mM HEPES pH 7.9, 420 mM NaCl, 25% (v/v) glycerol, 1.5 mM $MgCl_2$, 1 mM DTT, 0.2 mM EDTA containing protease and phosphatase inhibitor cocktails). Nuclear proteins were extracted by shaking for 30 min at 4°C. Cell fragments were removed by centrifugation (20,000 × g, 20 min, 4°C). Both fractions were combined for immunoprecipitation using the Pierce Magnetic HA-Tag IP/Co-IP Kit (Thermo Fisher Scientific) according to the manufacturer's acidic elution protocol. Eluates were analyzed by SDS-PAGE and silver staining using Pierce Silver Stain for Mass Spectrometry (Thermo Fisher Scientific) and interacting proteins were identified by LC-MS/MS.

Protein concentrations in the eluates were determined using the Pierce BCA protein assay kit (Thermo Fisher Scientific) against a bovine serum albumin (BSA) standard curve. We digested 25 µg of protein per bait sample using trypsin according to the FASP protocol [9]. After overnight digestion, samples were acidified with 1% (v/v) trifluoroacetic acid (TFA). A peptide sample aliquot corresponding to 5 µg of digested protein was desalted using self-packed StageTips [10]. Desalted samples were dried in a vacuum centrifuge and stored at –80°C. Excised silver-stained gel bands were destained and digested with trypsin [11], without reduction and alkylation of cysteines. Extracted peptides were acidified, desalted with StageTips and stored as described above. LC-MS/MS analysis was carried out using an Ultimate 3000 nanoLC (Thermo Fisher Scientific) coupled via a nanospray interface to a Q Exactive Plus mass spectrometer (Thermo Fisher Scientific).

Prior to LC-MS/MS analysis, samples were reconstituted in 2% (v/v) acetonitrile/0.05% (v/v) TFA to a (theoretical) concentration of 0.5 µg/µL. Samples (2 µL) were loaded on a trap column (C18, Acclaim PepMap 100, 300 µM × 5 mm, 5 µm particle size, 100 Å pore size; Thermo Fisher Scientific) at a flow rate of 10 µL/min for 3 min using 2% (v/v) acetonitrile/0.05% (v/v) TFA in ultrapure water. The peptides were separated on a reversed-phase column (C18, Acclaim Pepmap C18, 75 µm × 50 cm, 2 µm particle size, 100 Å pore size; Thermo Fisher Scientific) at a flow rate of 250 nL/min. Eluents were composed of 0.1% (v/v) formic acid in ultrapure water (A) and 80% (v/v) acetonitrile/0.1% (v/v) formic acid in ultrapure water (B). The following gradient was applied: 2.5–18% B over 60 min, 18–35% B over 40 min, 35–99% B over 5 min, 99% B for 20 min. The mass spectrometer was operated in positive ion mode. MS full scans (MS1, *m/z* 350–1400) were acquired at a resolution of 70,000 (FWHM, at *m/z* 200) with internal lock mass calibration on *m/z* 445.120025. The AGC target and maximum injection time were set to 3×10^6 and 50 ms, respectively. For MS^2, the 12 most intense ions with charge states 2–4 were fragmented by higher-energy c-trap dissociation (HCD) at 27% normalized collision energy. Dynamic exclusion was set to "auto" (chromatographic peak width 15 s) with a precursor tolerance of 5 ppm. MS^2 spectra were recorded at a resolution of 17,500. The AGC target was 5×10^4, the minimum AGC target was 5×10^2, the maximum injection time was 50 ms, and the precursor isolation window was 1.5 *m/z*.

After in-gel digestion, dried peptides were dissolved in 6 µL 2% (v/v) acetonitrile/0.05% (v/v) TFA and 2 µL was loaded on a trap column. Samples were analyzed as described above, with the following modifications: the AGC target minimum and maximum injection time for MS^2 were set to 5.5×10^2 and 55 ms, respectively. Ions with charged states 2–5 were fragmented. The gradient for peptide separation was programmed as follows: 2.5–45% B over 40 min, 45–99% B over 5 min, 99% B for 20 min.

Database searching and label-free quantification were carried out in Proteome Discoverer v2.2 (Thermo Fisher Scientific). Spectral files were searched using SequestHT against a *D. melanogaster* protein list (UniProt proteome: AUP000000803, downloaded 2018-06–26), supplemented with a list of common contaminants (cRAP, https://www.thegpm.org/crap/) and the polypeptide sequence of recombinant EGFP-NtFT4. Precursor and fragment mass tolerances were set to 10 ppm and 0.02 Da, respectively. The minimum peptide length was six and a maximum of two missed cleavages was allowed. Methionine oxidation and *N*-acetylation of protein N-termini were set as variable modifications. In the case of FASP-digested samples, carbamidomethylation of cysteines was set as a static

modification. Peptide spectrum matches (PSMs) were filtered using the Percolator node to satisfy a false discovery rate (FDR) of 0.01 (based on *q*-values). Subsequently, identifications were filtered to achieve a peptide and protein level FDR of 0.01. MS^1 features were determined using the Minora node with default settings. LC-MS/MS runs were chromatographically aligned with a maximum retention time drift of 10 min. Protein ratios were calculated as the median of all possible pairwise ratios of connected unique and razor peptides.

Flow cytometry gating strategy and FRET analysis

Forward versus side scatter (FSC vs. SSC) plots were used to define intact cells, and doublets were excluded by plotting the height versus area of FSC for subsequent FRET analysis. Fluorescence emission was detected by excitation at 405 nm using bandpass (BP) filters 450/40 nm (donor emission) and 525/50 nm (FRET emission) and excitation at 488 nm using the BP filter 530/30 nm (acceptor emission). The gates were uniformly applied to all experiments and different negative controls (nontransfected cells, all constructs as single transfections, and all constructs in combination with unfused Cer or unfused EYFP, respectively) were included to ensure gate stringency (Supplementary Figure 8). Data were analyzed using Flowing Software v2.5.1 and relative FRET efficiency was calculated from gate 4 (plot FRET vs. Donor, Supplementary Figure 8) for three independent samples for each combination.

REFERENCES

1. Post J, van Deenen N, Fricke J, Kowalski N, Wurbs D, Schaller H, Eisenreich W, Huber C, Twyman RM, Prüfer D, Gronover CS. Laticifer-specific cis-prenyltransferase silencing affects the rubber, triterpene, and inulin content of Taraxacum brevicorniculatum. Plant Physiol. 2012; 158:1406–17. https://doi.org/10.1104/pp.111.187880 PMID:22238421

2. Bevan M. Binary Agrobacterium vectors for plant transformation. Nucleic Acids Res. 1984; 12:8711–21. https://doi.org/10.1093/nar/12.22.8711 PMID:6095209

3. Töpfer R, Matzeit V, Gronenborn B, Schell J, Steinbiss HH. A set of plant expression vectors for transcriptional and translational fusions. Nucleic Acids Res. 1987; 15:5890. https://doi.org/10.1093/nar/15.14.5890 PMID:3615207

4. Bischof J, Maeda RK, Hediger M, Karch F, Basler K. An optimized transgenesis system for Drosophila using germ-line-specific phiC31 integrases. Proc Natl Acad Sci U S A. 2007; 104:3312–7. http://www.pnas.org/cgi/doi/10.1073/pnas.0611511104 https://doi.org/10.1073/pnas.0611511104 PMID:17360644

5. Horsch RB, Klee HJ, Stachel S, Winans SC, Nester EW, Rogers SG, Fraley RT. Analysis of Agrobacterium tumefaciens virulence mutants in leaf discs. Proc Natl Acad Sci U S A. 1986; 83:2571–5. https://doi.org/10.1073/pnas.83.8.2571 PMID:3458219

6. Hoekema A, Hirsch PR, Hooykaas PJJ, Schilperoort RA. A binary plant vector strategy based on separation of *vir*- and T-region of the *Agrobacterium tumefaciens* Ti-plasmid. Nature. 1983; 303:179–80. https://doi.org/10.1038/303179a0

7. Clough SJ, Bent AF. Floral dip: a simplified method for Agrobacterium-mediated transformation of Arabidopsis thaliana. Plant J. 1998; 16:735–43. https://doi.org/10.1046/j.1365-313x.1998.00343.x PMID:10069079

8. Walter M, Chaban C, Schütze K, Batistic O, Weckermann K, Näke C, Blazevic D, Grefen C, Schumacher K, Oecking C, Harter K, Kudla J. Visualization of protein interactions in living plant cells using bimolecular fluorescence complementation. Plant J. 2004; 40:428–38. https://doi.org/10.1111/j.1365-313X.2004.02219.x PMID:15469500

9. Wiśniewski JR, Zougman A, Nagaraj N, Mann M. Universal sample preparation method for proteome analysis. Nat Methods. 2009; 6:359–62. https://doi.org/10.1038/nmeth.1322 PMID:19377485

10. Rappsilber J, Mann M, Ishihama Y. Protocol for micro-purification, enrichment, pre-fractionation and storage of peptides for proteomics using StageTips. Nat Protoc. 2007; 2:1896–906. https://doi.org/10.1038/nprot.2007.261 PMID:17703201

11. Shevchenko A, Tomas H, Havlis J, Olsen JV, Mann M. In-gel digestion for mass spectrometric characterization of proteins and proteomes. Nat Protoc. 2006; 1:2856–60. https://doi.org/10.1038/nprot.2006.468 PMID:17406544

12. Waterhouse A, Bertoni M, Bienert S, Studer G, Tauriello G, Gumienny R, Heer FT, de Beer TAP, Rempfer C, Bordoli L, Lepore R, Schwede T. SWISS-MODEL: homology modelling of protein structures and complexes. Nucleic Acids Res. 2018; 46:W296–303. https://doi.org/10.1093/nar/gky427 PMID:29788355
