# Supplementary material for: The tobacco phosphatidylethanolamine-binding protein NtFT4 increases the lifespan of Drosophila melanogaster by interacting with the proteostasis network
Source: Aging (Albany NY). 2022 Apr 8;14(7):2989–3029. doi: 10.18632/aging.204005 (PMC9037272; doi:10.18632/aging.204005)
Supplement: Supplementary Figures [file aging-14-204005-s002.pdf]

SUPPLEMENTARY FIGURES

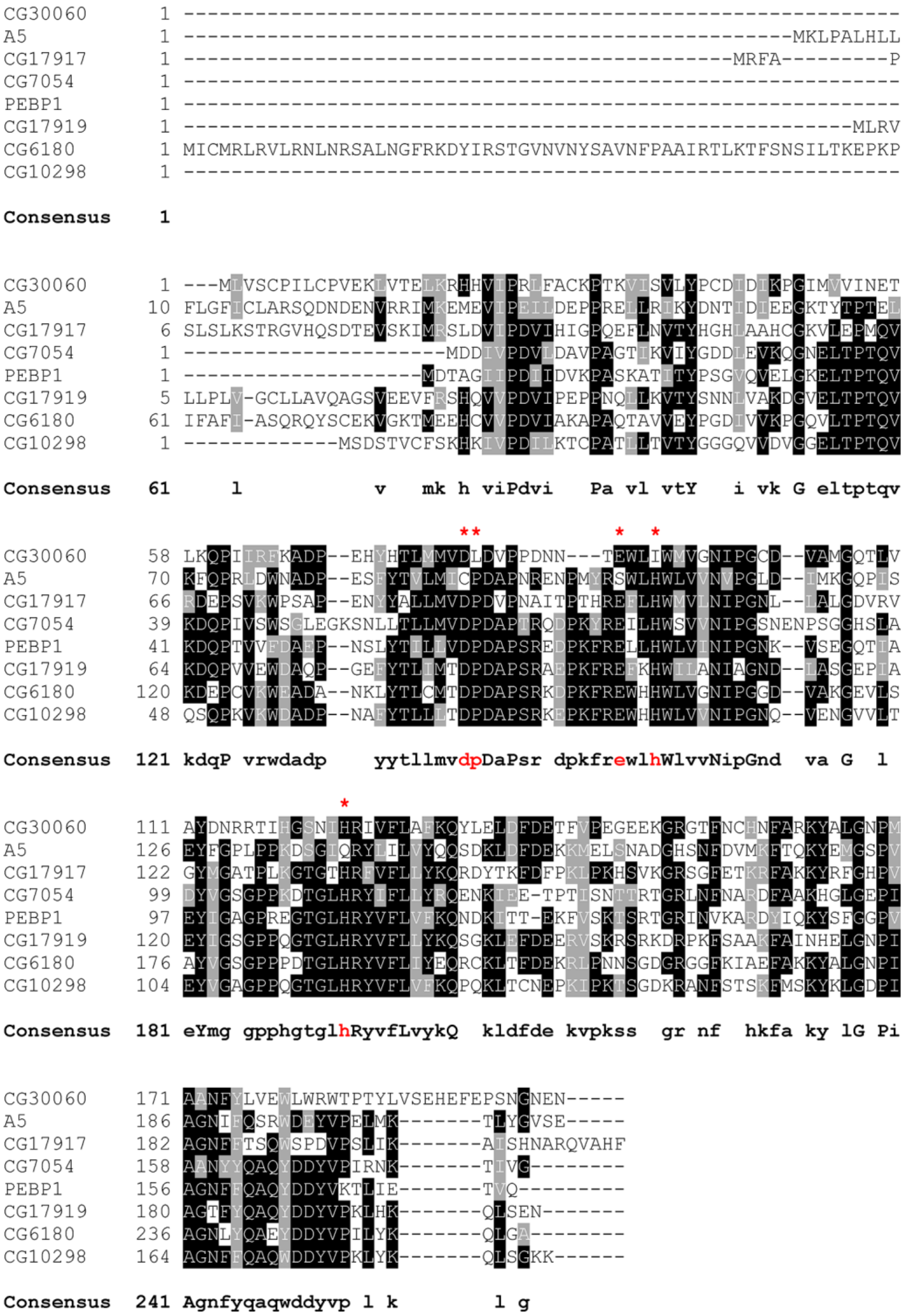

Supplementary Figure 1. Protein sequence alignment of the eight Drosophila PEBP-like proteins. Alignment of CG30060 (NP_725293.1), A5 (NP_476998.1), CG17917 (NP_649642.1), CG7054 (NP_651050.1), PEBP1 (NP_651051.1), CG17919 (NP_649644.1), CG6180 (NP_609588.1) and CG10298 (NP_649643.1) using Clustal Omega (https://www.ebi.ac.uk/Tools/msa/clustalo/). Box shading represents identical amino acids (black) and similar amino acids (gray), with at least 50% of the sequences carrying the corresponding amino acids (BOXSHADE v3.21). Red letters and asterisks indicate variations in the conserved motifs of the phosphatidylethanolamine-binding pocket in proteins A5 and CG30060.

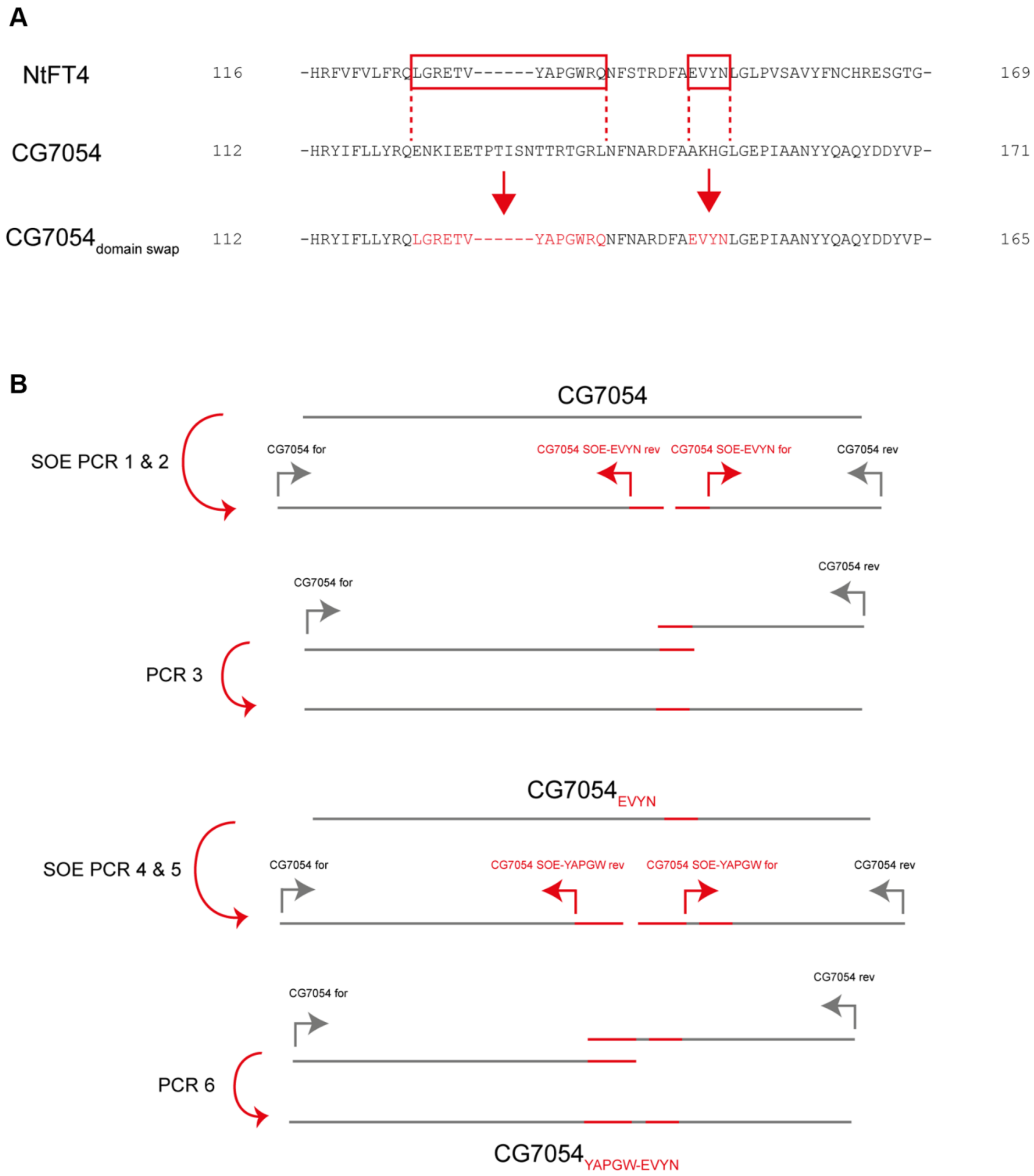

Supplementary Figure 2. Splice overlap extension (SOE)-PCR scheme for the creation of the CG7054-DS sequence encoding a chimeric CG7054 protein with NtFT4 domains. (**A**) Alignment of the CG7054 and NtFT4 segments that were exchanged in CG7054-DS. Motifs that are necessary for floral activators (NtFT4, red boxes) were used to replace the corresponding region of CG7054, allowing the expression in tobacco of an animal PEBP which contains conserved motifs for floral transition (red letters). (**B**) Steps and primers used to introduce segments of tobacco NtFT4 into Drosophila CG7054 by SOE-PCR. Red parts represent overhangs added during SOE-PCR steps 1 and 2, and steps 4 and 5, which subsequently align in the fragment templates for steps 3 and 6 to generate the full-length CG7054-DS ($CG7054_{YAPGW\text{-}EVYN}$).

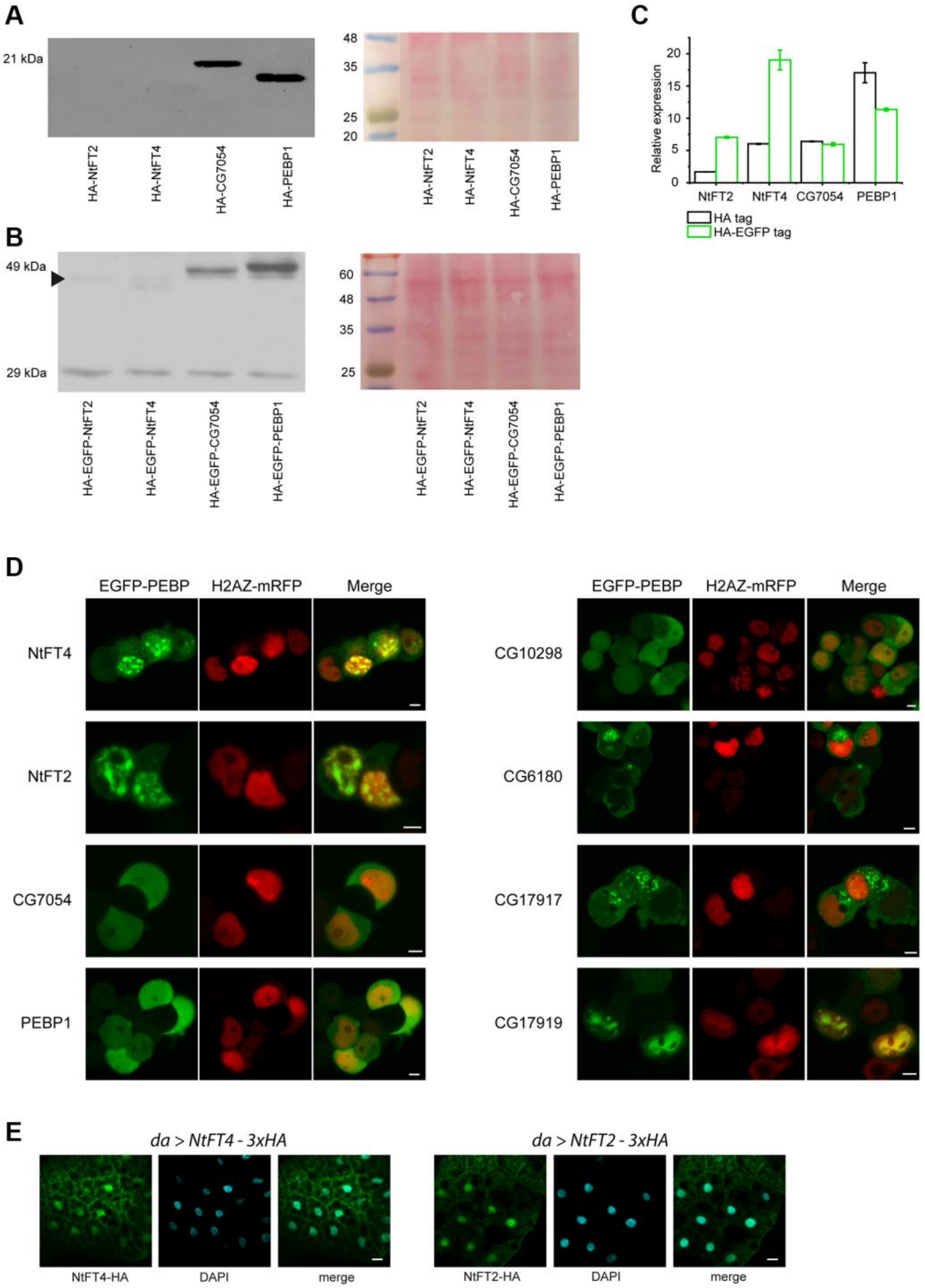

Supplementary Figure 3. Transient expression of tagged PEBPs in S2 and HEK-293T cells. (**A**) Western blot of transiently expressed HA-NtFT2, HA-NtFT4, HA-CG7054 and HA-PEBP1 in S2 cells. HA-tagged proteins were detected using a rabbit anti-HA antibody and comparable protein loading and transfer were confirmed by staining with Ponceau S. (**B**) Western blot of transiently expressed HA-EGFP-NtFT2, HA-EGFP-NtFT4, HA-EGFP-CG7054 and HA-EGFP-PEBP1 in S2 cells. HA-EGFP-tagged proteins were detected using a rabbit anti-HA antibody and comparable protein loading and transfer were tested by staining with Ponceau S. Cleaved HA-EGFP was also detected at ~29 kDa. The weak bands representing HA-EGFP-NtFT2 and the adjacent HA-EGFP-NtFT4 are indicated by the arrowhead. (**C**) Expression levels were simultaneously determined by quantitative RT-PCR. Relative expression levels were calculated for HA (black) and HA-EGFP (green) fusion constructs in relation to *Gapdh2*. Data are means ± SEM (n = 3). (**D**) Confocal images showing the subcellular localization of EGFP-PEBP fusion proteins expressed in HEK-293T cells. The cells were transiently transfected with EGFP-PEBP (NtFT4, NtFT2, CG7054, PEBP1, CG10298, CG6180, CG17917, CG17919) and H2AZ-mRFP (red) constructs and analyzed 1 d post-transfection. Scale bar = 5 µm. (**E**) UAS-NtFT4-3xHA and UAS-NtFT2-3xHA flies were mated with the da-Gal4 driver strain to detect the expression of tobacco PEBPs NtFT4 and NtFT2 in Drosophila. Proteins were detected in fat body cells by immunostaining using an anti-HA mouse monoclonal antibody (green). Nuclei were counterstained with DAPI (blue). Scale bar = 20 µm.

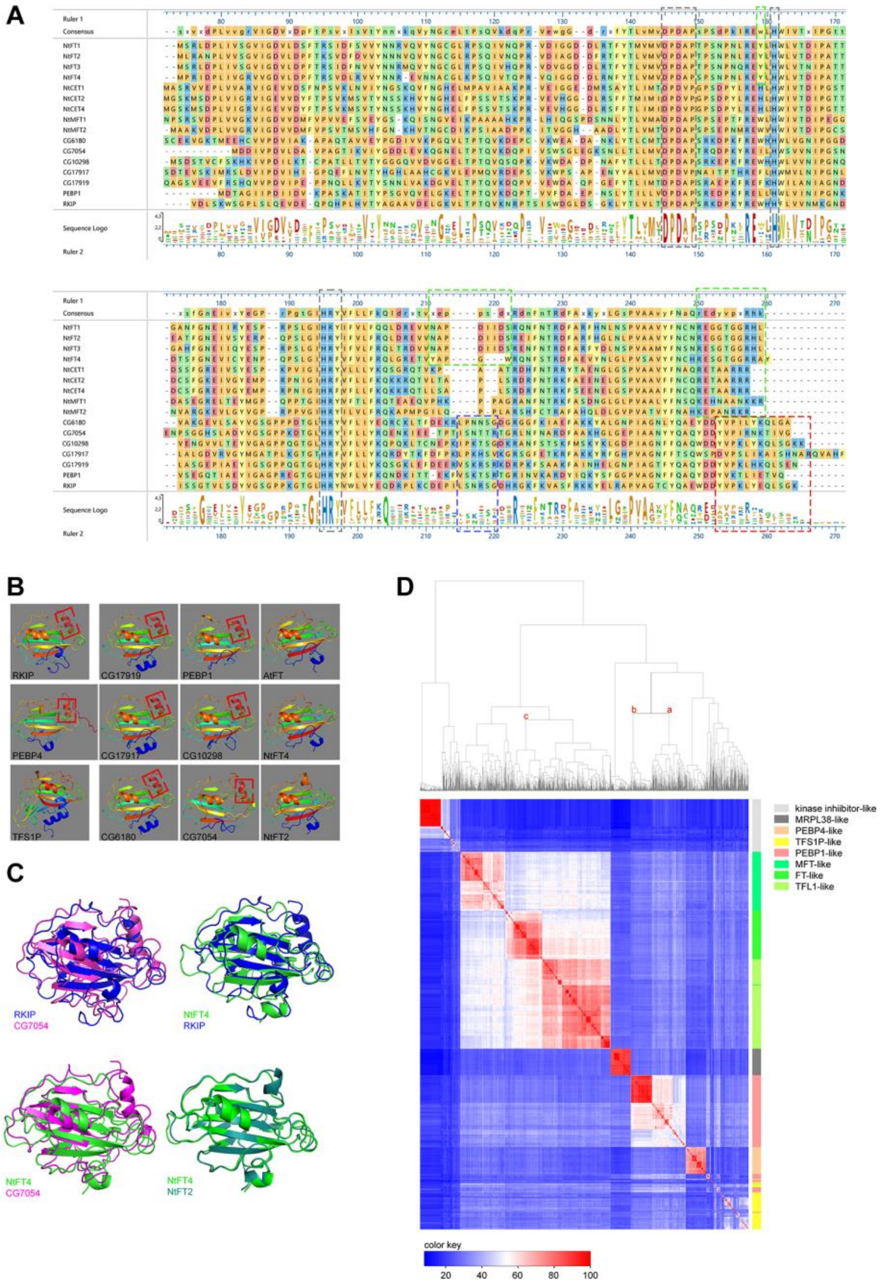

Supplementary Figure 4. Sequence and 3D structure of tobacco and Drosophila PEBPs. (**A**) Peptide sequences of NtFT1, NtFT2, NtFT3, NtFT4, NtCET1, NtCET2, NtCET4, NtMFT1, NtMFT2, CG6180, CG7054, CG10298, CG17917, CG17919, PEBP1 and human RKIP were aligned using MegAlign Pro (DNAStar) and Clustal Omega. Conserved amino acids are indicated by letter size in the sequence logo. Characteristic motifs are enclosed in dashed boxes (gray = conserved PEBP motifs, green = plant PEBP motifs of floral regulators, blue = major differences between animal and plant PEBPs in the loop region, red = C-terminal α helix of animal PEBPs). The alignment of all eight PEBP-like proteins from Drosophila, including A5 and CG30060, is shown in Supplementary Figure 11. (**B**) The 3D protein structures of human RKIP and PEBP4, yeast TFS1P, Drosophila CG17919, CG17917, CG6180, PEBP1, CG10298, CG7054, Arabidopsis FT and tobacco NtFT4 and NtFT2. The crystal structures of RKIP, PEBP4, TFS1P, CG7054 and FT are known and the other PEBPs were predicted using Swiss-MODEL [12]. The red boxes indicate the C-terminal α-helix of animal PEBPs. Coloring indicates the N-terminus (blue) to the C-terminus (red). (**C**) Aligned 3D structures of RKIP (blue) with CG7054 (magenta) or NtFT4 (green) and of NtFT4 (green) with CG7054 (magenta) and NtFT2 (turquoise). (**D**) Heat map identity matrix of 1596 PEBPs from species ranging from prokaryotes to mammals and plants. PEBP sequences were aligned using Clustal Omega and the output identity matrix was plotted as a heat map using R Studio v1.3.1093. Color coding represents identities ranging from low (blue) to high (red) in percent identity. PEBPs were assigned to prokaryotic *kinase inhibitor-like* (light gray), to *TFS1P-like* (yellow), *MRPL38-like* (gray), *PEBP1-like* (light red), *PEBP4-like* (orange), plant *MFT-like* (blue-green), *TFL1-like* (yellow-green) and *FT-like* (green) indicated at the top. In the dendrogram *a* indicates the PEBP1 cluster, in which all Drosophila PEBPs can be found, *b* indicates the highly-conserved mammalian PEBP1-like proteins and *c* indicates the plant FT-like subgroup in which tobacco NtFT4 and NtFT2 are found.

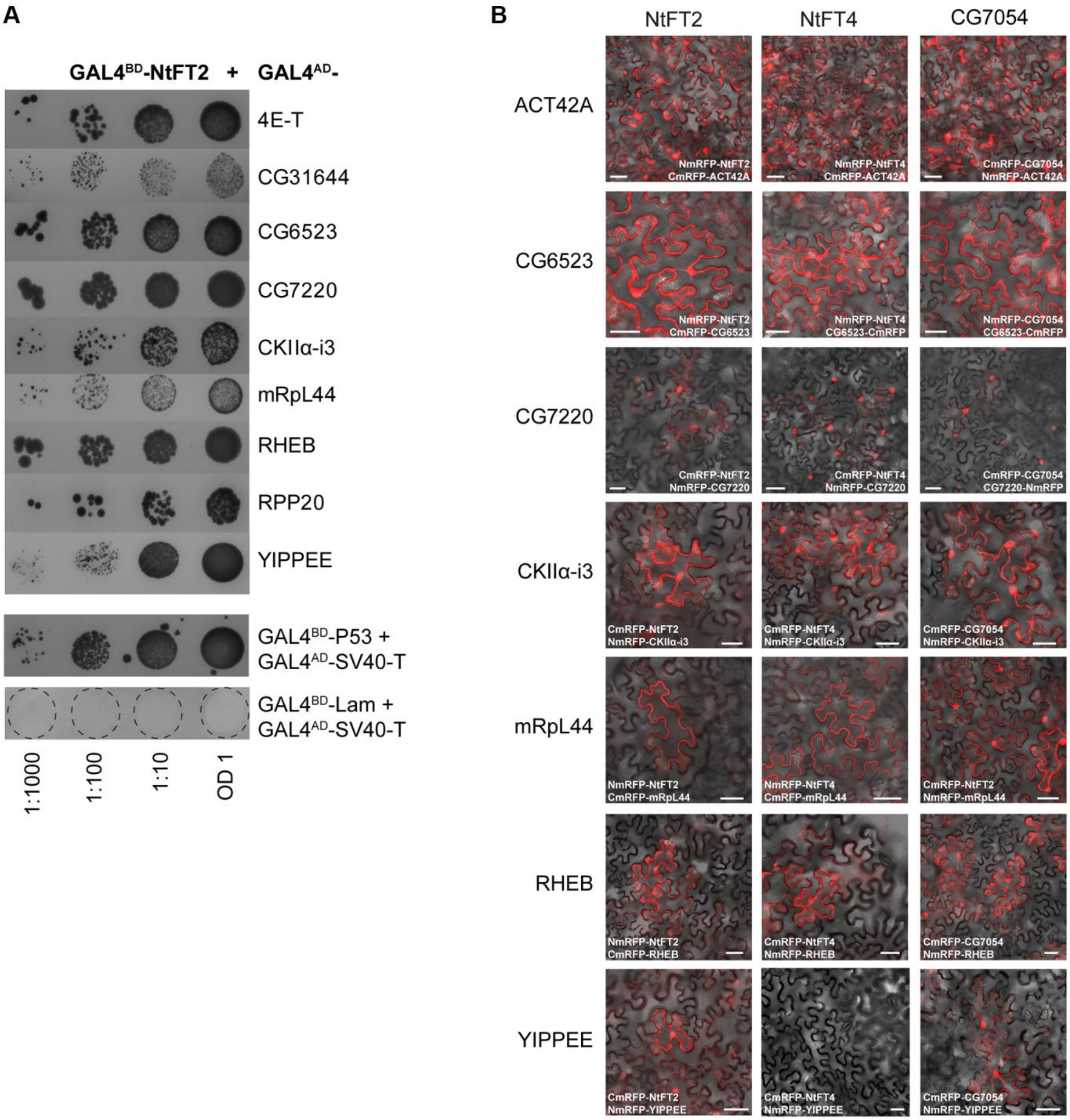

Supplementary Figure 5. Interaction partners of NtFT2, NtFT4 and CG7054 identified by yeast-two hybrid screening of a normalized Drosophila cDNA library. (**A**) Coding sequences of putative interaction partners fused to the Gal4 activation domain (Gal4^{AD}) and a bait construct comprising the Gal4 binding domain (Gal4^{BD}) fused to NtFT2 were simultaneously introduced into *S. cerevisiae* strain Y2HGold for drop tests on selective plates. The interaction of murine p53 with the large T-antigen (SV40-T) served as a positive control, whereas the combination of lamin with SV40-T served as a negative control. The different dilutions of yeast suspensions (undiluted, 1 :10, 1 :100 and 1 :1000) are indicated. The interaction of NtFT2 with Act42 was not tested due to growth defects of the prey strain expressing Act42A. The interaction with NtFT4 was not tested in yeast due to auto-activation of the bait strain expressing Gal4-BD-NtFT4. (**B**) Bimolecular fluorescence complementation (BiFC) in *N. benthamiana* leaf epidermal cells to confirm the interaction with NtFT2 in a different background and analyze the interaction with NtFT4 and CG7054. Representative merged bright-field and fluorescence images are shown and the corresponding split-mRFP constructs used for co-transformation are indicated. BiFC provided unclear results for the interactions with 4E-T and CG31644. Scale bars = 50 µm.

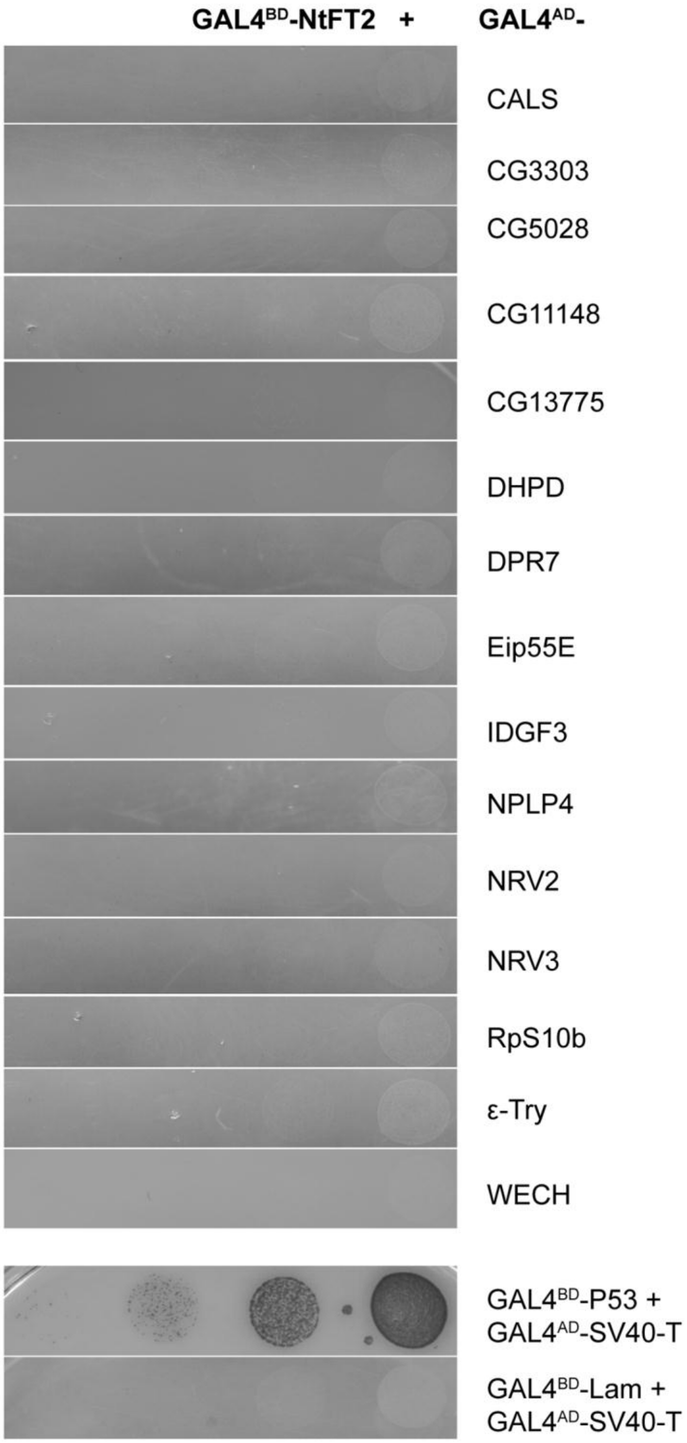

Supplementary Figure 6. Putative interaction partners of NtFT2 identified in the yeast two-hybrid screen which were not confirmed in drop tests with the full-length coding sequences. Coding sequences of putative interaction partners in fusion with the Gal4 activation domain (Gal4^{AD}) were introduced into *S. cerevisiae* Y2HGold cells with the bait construct comprising the Gal4 binding domain (Gal4^{BD}) fused to NtFT2 for drop tests on selective plates. The interaction of murine p53 with the large T-antigen (SV40-T) served as a positive control, and the combination of lamin with SV40-T served as a negative control. The different dilutions of yeast suspensions (undiluted, 1 :10, 1 :100 and 1 :1000) are indicated.

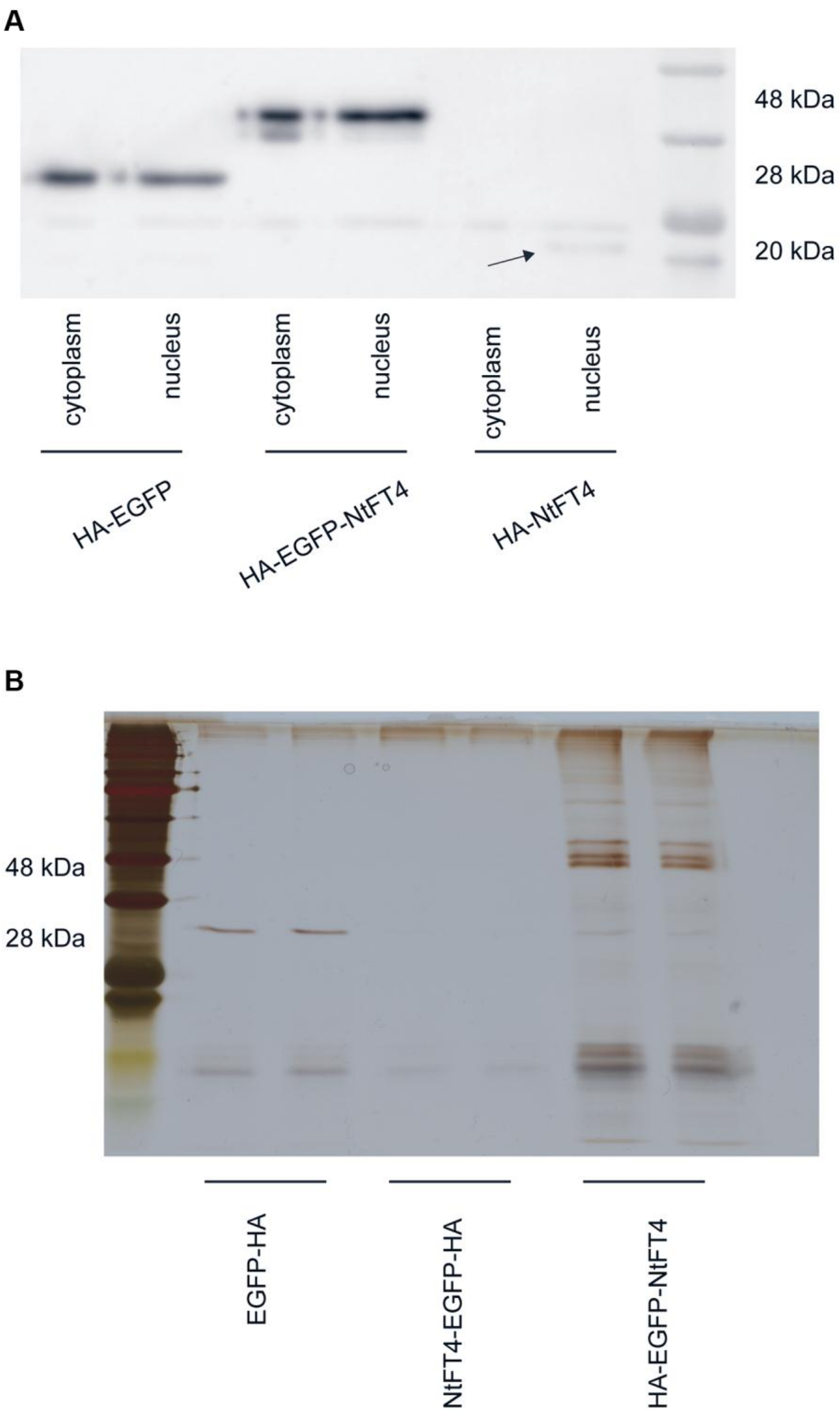

Supplementary Figure 7. Detection of tagged and codon-optimized NtFT4 expressed in S2 cells and immunoprecipitation to identify interaction partners in flies. (**A**) Different extraction protocols were tested to extract sufficient amounts of NtFT4 for immunoprecipitation. Western blot of transiently expressed HA-EGFP, HA-EGFP-NtFT4 and HA-NtFT4 in the cytoplasmic and nuclear protein fractions. The arrow indicates a weak band corresponding to HA-NtFT4 in the nuclear fraction. (**B**) Silver staining of eluates of different HA-tagged bait proteins after immunoprecipitation. Transiently expressed HA-EGFP-NtFT4, NtFT4-EGFP-HA and HA-EGFP were extracted in separate cytoplasmic and nuclear fractions as above and both fractions were combined for immunoprecipitation. Because eluates using NtFT4-EGFP-HA showed only traces of protein bands, these samples were not processed any further. Regions showing distinct bands in the eluates of HA-EGFP-NtFT4 were excised and both these gel pieces and the complete eluates were analyzed by LC-MS/MS compared to the corresponding samples of the HA-EGFP eluates.

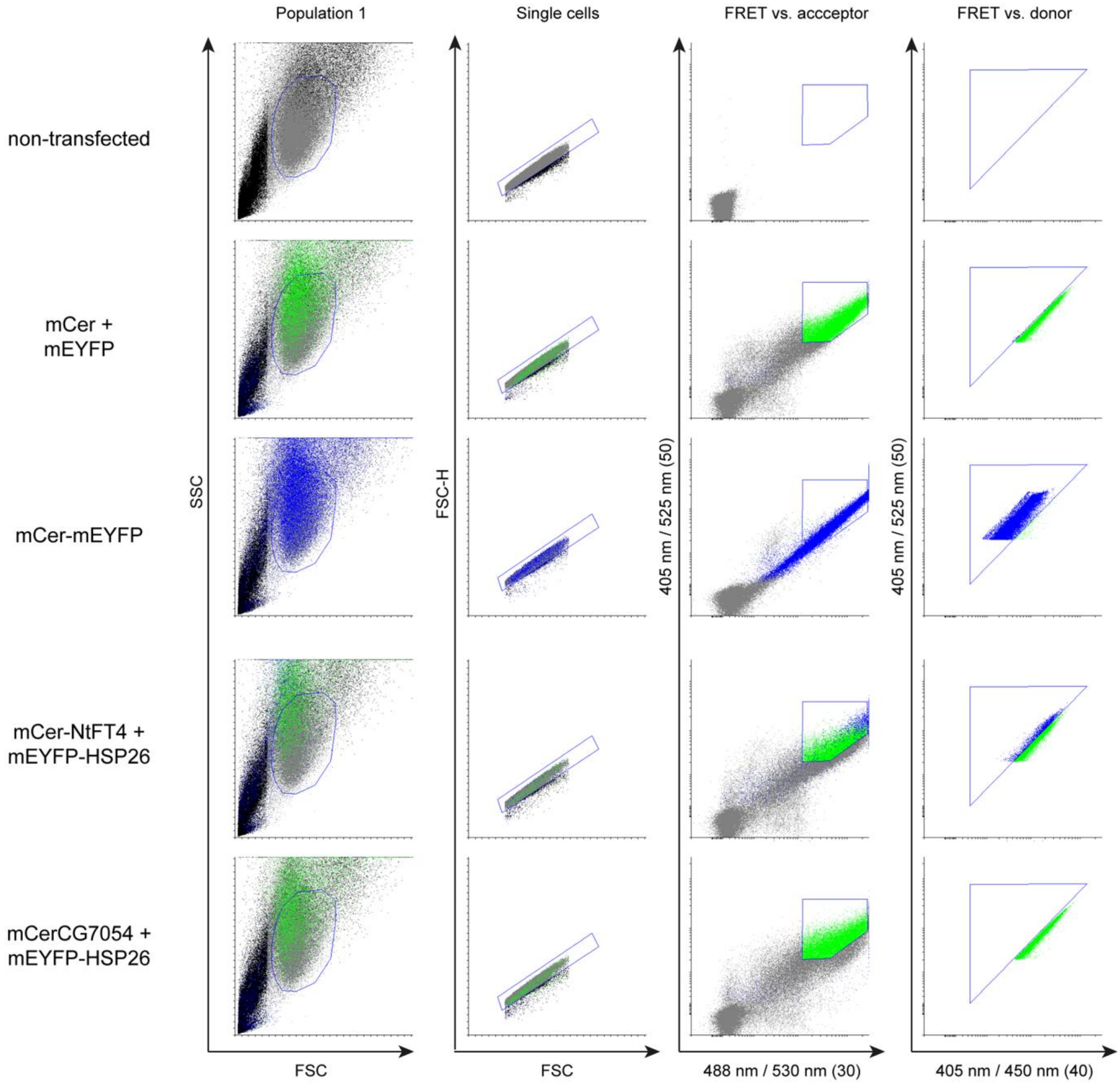

Supplementary Figure 8. Gating strategy for FRET analysis by flow cytometry. All samples were gated using the same settings and representative samples are shown. Single cells were gated using FSC and SSC to define intact cell population 1, and the area (FSC-A) and height (FSC-H) of FSC to exclude doublets. Single cells were then plotted for events identified by excitation at 405 nm and detection at 525 (50) nm against excitation at 488 nm and detection at 530 (30) nm (FRET vs. acceptor). The distinct population with emissions at both excitation wavelengths was then plotted for events identified by excitation at 405 nm and detection at 525 (50) nm against excitation at 405 nm and detection at 450 (40) nm (FRET vs. donor, quantification gate for FRET efficiency). The gate to quantify only FRET-positive cells was set using all negative controls included in the experiments expressing both fluorophores (Cer + EYFP, Cer-PEBP + EYFP, Cer + EYFP-POI), in which < 0.5% events of the parental gate could be detected. A protein fusion of mCer-mEYFP served as positive control with maximum FRET activity. Abbreviation: POI: protein of interest.

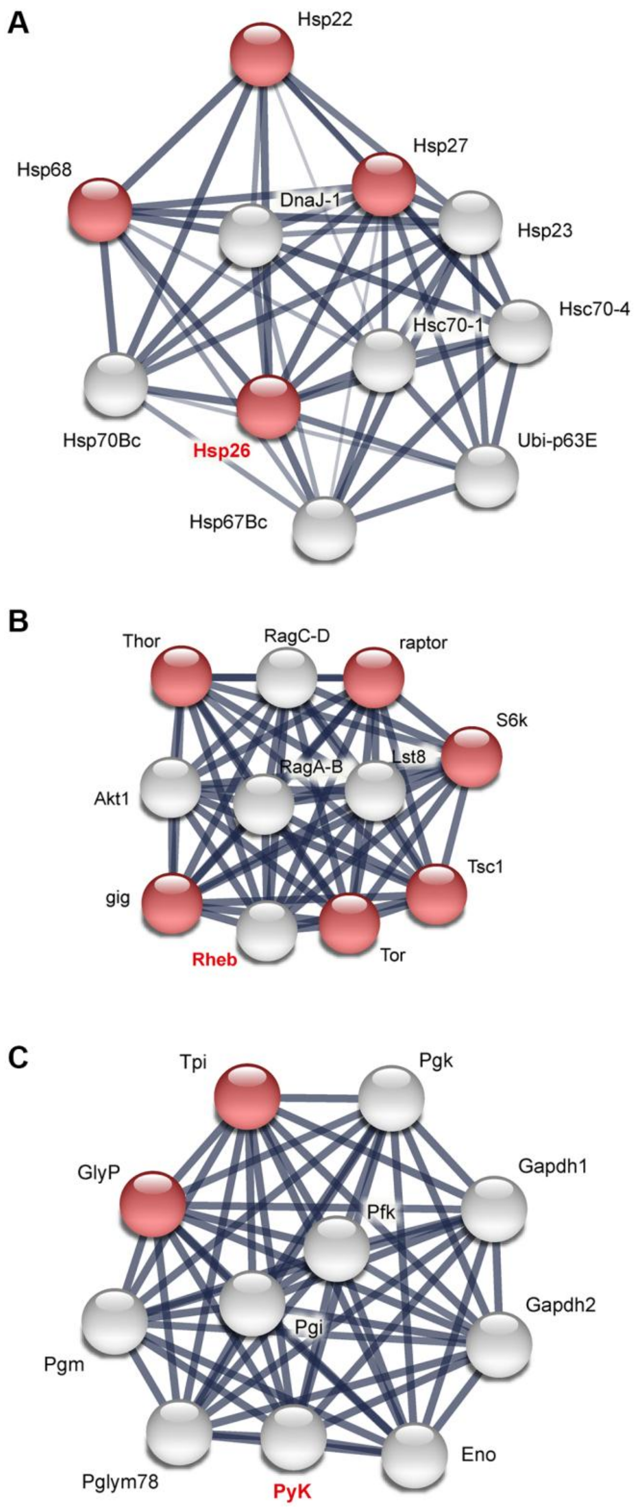

Supplementary Figure 9. Interaction networks of HSP26, RHEB and PyK, which are associated with lifespan determination. (**A**) Predicted interaction network of HSP26 in Drosophila. HSP26 is associated with several other heat shock proteins and HSP26 itself was shown to influence longevity in Drosophila. (**B**) Predicted interaction network of RHEB in Drosophila. RHEB is associated with several proteins in the IIS/TOR signaling pathway, which were shown to determine lifespan in different model organisms. RHEB itself is not yet directly linked to the determination of lifespan. (**C**) Predicted interaction network of PyK in Drosophila. PyK was shown to influence lifespan in nematodes [34] but is also likely to influence lifespan through triose phosphate isomerase (Tpi) and glycogen phosphorylase (GlyP). Red nodes indicate proteins related to the determination of lifespan by direct experimental evidence or if orthologs in other species were found to alter lifespan (https://string-db.org). Brightness and thickness of lines correspond to the confidence of an interaction.

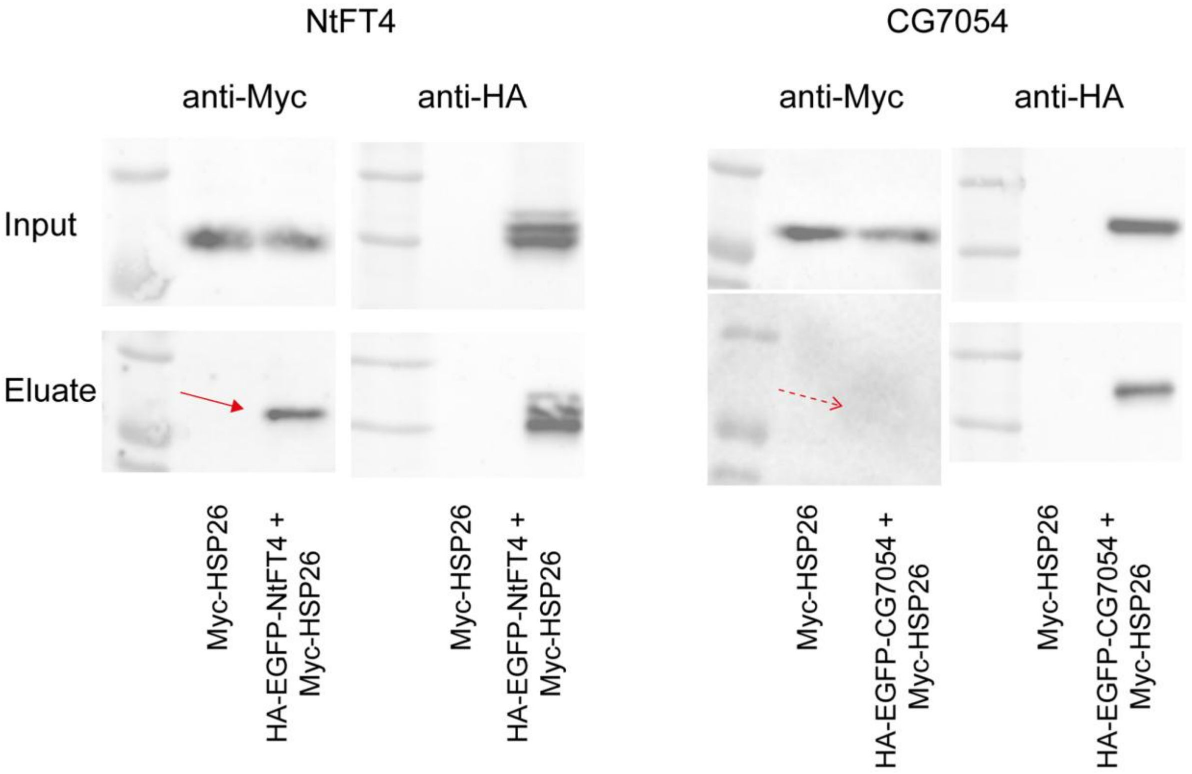

Supplementary Figure 10. Co-immunoprecipitation shows that HSP26 interacts with NtFT4 but not CG7054. A Myc-tagged HSP26 was transiently expressed with HA-EGFP-NtFT4 or HA-EGFP-CG7054 in S2 cells. Protein extracts from single transfections (Myc-HSP26) and double transfections (Myc-HSP26 with HA-EGFP-NtFT4 or HA-EGFP-CG7054) were precipitated using magnetic anti-HA beads and the eluates and the input extracts were analyzed by western blot using mouse anti-Myc and rabbit anti-HA antibodies. Myc-HSP26 was only detected in eluates using HA-EGFP-NtFT4 as the bait (solid arrow), whereas no band was detected in eluates using HA-EGFP-CG7054 as the bait (dashed arrow).

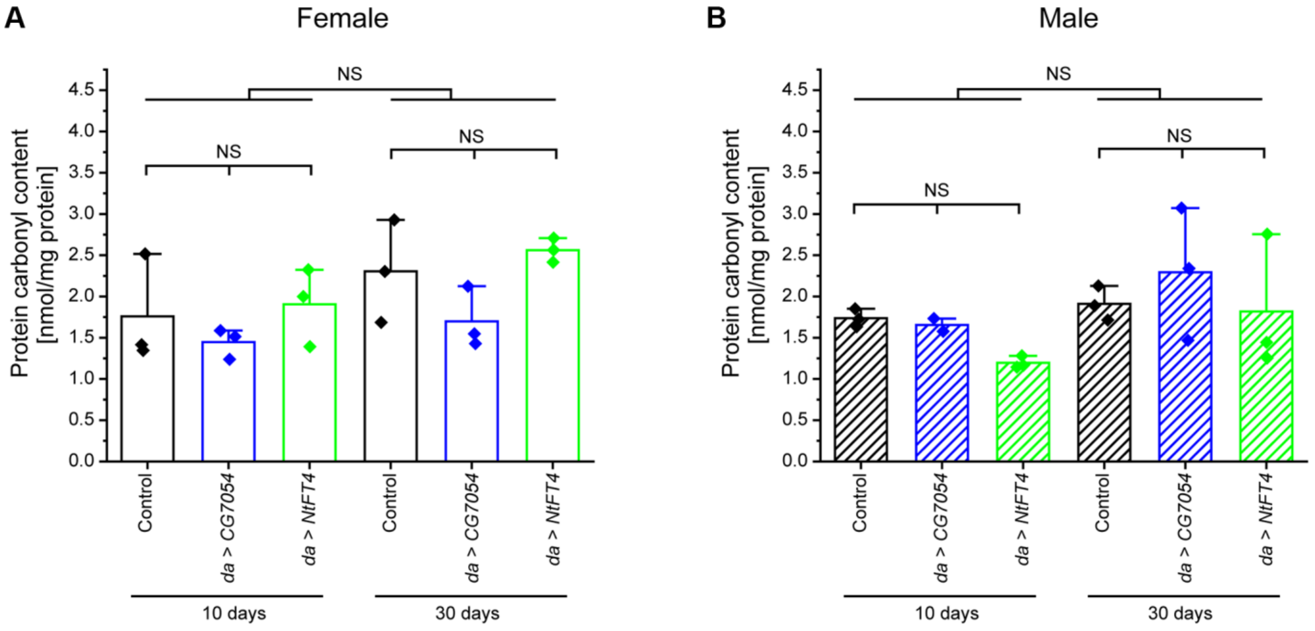

Supplementary Figure 11. Protein carbonylation in *da > CG7054* and *da > NtFT4* flies. Carbonyl content in protein extracts of female (**A**) and male (**B**) flies (10 or 30 days old) expressing CG7054 or NtFT4 after mating UAS-NtFT4 or UAS-CG7054 with the *da-Gal4* driver strain compared with *da-Gal4* x Oregon-R (control). Data are means ± SEM (*n* = 3, except for *da > CG7054* ♂10 days). Significance was tested by one-way ANOVA and Tukey's post hoc test. Abbreviations: *NS*: not significant.

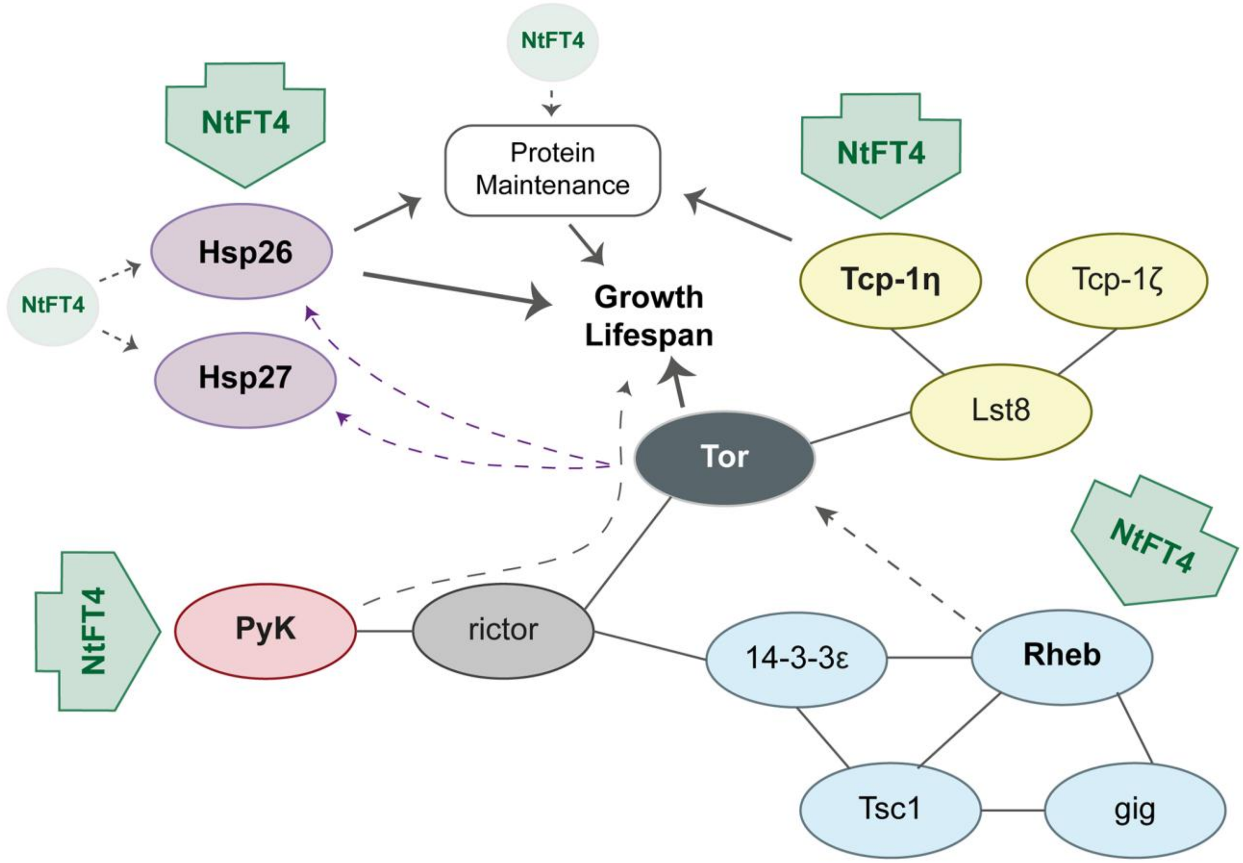

Supplementary Figure 12. Overview of NtFT4 interactions that intersect with IIS/TOR signaling in Drosophila. Physical interactions between NtFT4 and RHEB, CCT7 (Tcp-1η), PyK and HSP26 are indicated by green arrows. HSP26 and PyK have immediate (solid arrow) or indirect (dashed arrow) functions in the determination of lifespan. RHEB and CCT7 act through their association with TOR. Physical or genetic interactions with components of the IIS/TOR network are indicated by solid lines. NtFT4 also influences the regulation of *Hsp26* and *Hsp27* gene expression (NtFT4 in green circles) and of proteases which could contribute to protein maintenance and homeostasis. Protein maintenance is also influenced by CCT7 and the heat shock proteins.
