# Supplementary material for: The tobacco phosphatidylethanolamine-binding protein NtFT4 increases the lifespan of Drosophila melanogaster by interacting with the proteostasis network
Source: Aging (Albany NY). 2022 Apr 8;14(7):2989–3029. doi: 10.18632/aging.204005 (PMC9037272; doi:10.18632/aging.204005)
Supplement: Supplementary Tables 1-4 [file aging-14-204005-s003.pdf]

SUPPLEMENTARY TABLES

Supplementary Table 1. Survival of male flies with dysregulated PEBP expression [*da-Gal4/UAS-CG7054*, *da-Gal4/UAS-NtFT2* or *da-Gal4/UAS-NtFT4*)] or [*da-Gal4/UASt-CG7054^{dsRNA}*] compared with the control +/*da-Gal4*.

	median lifespan [d]	25 % estimate [d]	mean lifespan [d]	Equality vs. Control (χ^2)	Equality vs. CG7054 (χ^2)	Equality vs. NtFT2 (χ^2)
**Control**	42	40	39.83 (± 0.53)	-	-	-
**CG7054**	47	40	43.28 (± 0.66)	55.51 ($p = 9.31 \times 10^{-14}$)	-	-
**CG7054^{dsRNA}**	25	7	19.95 (± 0.83)	330.66 ($p = 0$)	351.66 ($p = 0$)	-
**NtFT2**	40	30	36.93 (± 0.93)	1.05 ($p = 0.31$)	11.99 ($p = 5.36 \times 10^{-4}$)	-
**NTFT4**	47	37	42.7 (± 0.67)	41.22 ($p = 1.36 \times 10^{-10}$)	0.008 ($p = 0.93$)	10.98 ($p = 9.19 \times 10^{-4}$)

Median lifespans, 25% quartile estimates and mean lifespans were calculated based on Kaplan-Meier survival curves, and χ^2 and *p*-values were calculated using the Mantel-Cox method.

Supplementary Table 2. Overview of NtFT4 putative interaction partners identified by immunoprecipitation with HA-EGFP-NtFT4.

Name	Abundance ratio (HA-EGFP-NtFT4 / HA-EGFP)	Confirmed (method)
Cbs	100.0	no
CCT2	100.0	yes (Co-IP)
CCT3	100.0	no
CCT5	100.0	no
CCT6	100.0	no
CCT7	100.0	yes (Co-IP, FRET)
Inos	100.0	no
p47	100.0	yes (Co-IP)
Pen	100.0	yes (Co-IP, FRET)
Stip1	100.0	no
tudor-SN	100.0	yes (Co-IP, FRET)
Hsp26	71.4	yes (Co-IP, FRET)
Rack1	28.1	no
Df31	21.3	yes (BiFC)
eEF1beta	16.1	no
PyK	14.7	yes (Co-IP, FRET)
14-3-3zeta	13.0	no
CG4364	7.4	yes (Co-IP, FRET)
CG32549*	100.0	nd
Akap200*	100.0	nd

SerRS*	100.0	nd
CG12128*	100.0	nd
AspRS*	100.0	nd

HA-EGFP-NtFT4 and HA-EGFP were transiently expressed in S2 cells and nuclear and cytoplasmic proteins were precipitated using magnetic anti-HA beads. Eluted proteins were analyzed by LC-MS/MS and the protein abundance ratio was calculated by comparing HA-EGFP-NtFT4 and HA-EGFP eluates. An abundance ratio of 100 was specified if no peptides corresponding to this protein were detected in the HA-EGFP eluate. Protein interactions were also analyzed by co-immunoprecipitation in co-transfected S2 or HEK-293T cells (Co-IP) or by fluorescence resonance energy transfer (FRET) analysis of transfected HEK-293T cells. *Putative interaction partners that were not analyzed (nd).

Supplementary Table 3. Correlation analysis of expression profiles of female *da-Gal4* and *da > NtFT4* flies.

Sample		***da-Gal4***			***da > NtFT4***		
		**Pool 1**	**Pool 2**	**Pool 3**	**Pool 1**	**Pool 2**	**Pool 3**
da-Gal4	**Pool 1**	***1.000***	0.982	0.983	*0.974*	*0.973*	0.978
	**Pool 2**	0.982	***1.000***	0.995	0.983	0.996	0.997
	**Pool 3**	0.983	0.995	***1.000***	0.981	0.992	0.994
da > NtFT4	**Pool 1**	*0.974*	0.983	0.981	***1.000***	0.982	0.987
	**Pool 2**	*0.973*	0.996	0.992	0.982	***1.000***	0.998
	**Pool 3**	0.978	0.997	0.994	0.987	0.998	***1.000***

Similarity of the expression profiles of samples was determined by calculating Pearson's correlation coefficient r using GeneSpring GX v13.1. The correlation coefficients of all possible comparisons range from 0.973 to 0.998. The high correlation between all samples suggests that their global gene expression profiles may be very similar. The color range shows correlation coefficients from the lowest value of r ~ 0.97 (Italic) through to r = 1.00 (Bold Italic).

Supplementary Table 4. Number of deregulated genes in female flies expressing NtFT4 (*da-Gal4 > UAS-NtFT4*) vs. control flies (*da-Gal4*).

	**FC > 1.5 *p* < 0.05**	**FC > 1.5**
**Upregulated**	49	135
**Downregulated**	100	208
**Total**	149	343

Female flies aged 1, 5 and 10 days were pooled for whole-transcriptome analysis and flies expressing *NtFT4* were compared to control flies (*da-Gal4* x Oregon-R) using GeneChip Drosophila Genome 2.0 arrays (Affymetrix). Differential gene expression was calculated by pairwise comparison of averaged normalized signal values (n = 3). All genes with a fold-change (FC) > 1.5 and a p-value < 0.05 (t-test) are listed in the second column, and all genes with a FC > 1.5 including non-significant events are listed in the third column. A total of 18,952 probes was analyzed on the GeneChip.
